# Effective Leadership of Surgical Teams: A Mixed Methods Study of Surgeon Behaviors and Functions

**DOI:** 10.1016/j.athoracsur.2017.01.021

**Published:** 2017-08

**Authors:** Juliana L. Stone, Emma-Louise Aveling, Molly Frean, Morgan C. Shields, Cameron Wright, Francesca Gino, Thoralf M. Sundt, Sara J. Singer

**Affiliations:** a Department of Health Research and Policy, Harvard T. H. Chan School of Public Health, Boston, Massachusetts; b Cambridge Centre for Health Services Research, University of Cambridge, Cambridge, United Kingdom; c Health Care Management Department, The Wharton School, University of Pennsylvania, Philadelphia, Pennsylvania; d Institute for Behavioral Health, The Heller School for Social Policy and Management, Brandeis University, Waltham, Massachusetts; e Department of Surgery, Massachusetts General Hospital, Boston, Massachusetts; f Negotiation, Organizations & Markets, Harvard Business School, Boston, Massachusetts

## Abstract

**Background:**

The importance of effective team leadership for achieving surgical excellence is widely accepted, but we understand less about the behaviors that achieve this goal. We studied cardiac surgical teams to identify leadership behaviors that best support surgical teamwork.

**Methods:**

We observed, surveyed, and interviewed cardiac surgical teams, including 7 surgeons and 116 team members, from September 2013 to April 2015. We documented 1,926 surgeon/team member interactions during 22 cases, coded them by behavior type and valence (ie, positive/negative/neutral), and characterized them by leadership function (conductor, elucidator, delegator, engagement facilitator, tone setter, being human, and safe space maker) to create a novel framework of surgical leadership derived from direct observation. We surveyed nonsurgeon team members about their perceptions of individual surgeon's leadership effectiveness on a 7-point Likert scale and correlated survey measures with individual surgeon profiles created by calculating percentage of behavior types, leader functions, and valence.

**Results:**

Surgeon leadership was rated by nonsurgeons from 4.2 to 6.2 (mean, 5.4). Among the 33 types of behaviors observed, most interactions constituted elucidating (24%) and tone setting (20%). Overall, 66% of interactions (range, 43%–84%) were positive and 11% (range, 1%–45%) were negative. The percentage of positive and negative behaviors correlated strongly (*r* = 0.85 for positive and *r* = 0.75 for negative, *p* < 0.05) with nonsurgeon evaluations of leadership. Facilitating engagement related most positively (*r* = 0.80; *p* = 0.03), and negative forms of elucidating, ie, criticism, related most negatively (*r* = –0.81; *p* = 0.03).

**Conclusions:**

We identified 7 surgeon leadership functions and related behaviors that impact perceptions of leadership. These observations suggest actionable opportunities to improve team leadership behavior.

The Supplemental Material can be viewed in the online version of this article [http://dx.doi.org/10.1016/j.athoracsur.2017.01.021] on http://www.annalsthoracicsurgery.org.

Effective teamwork is essential to safe surgical care [1]. Nontechnical aspects of team performance, such as communication failures, contribute to surgical errors and adverse outcomes, especially in cardiac operations [2] and may be avoidable through improved interpersonal interactions [3]. Although research suggests that leadership impacts team performance [4], little is known about which leadership behaviors benefit surgical teamwork and which do not.

Surgeons are de facto team leaders, yet surgical training focuses on technical skills. Leadership behaviors are “picked up” by observing role models without evidence to support or refute their effectiveness in promoting team performance. An objective understanding of the impact of specific behaviors is therefore critical to optimizing surgical leadership.

We undertook an observational study of how surgeons actually lead in the operating room and created a tool for assessing surgeons’ leadership. Using data from surgical observations and interviews with team members, we characterized behaviors as positive, neutral, and negative and compared these with measures of surgeons’ leadership as perceived by surgical team members. In doing so, we developed a novel and empirically based framework of leadership functions and behaviors that can be used to enhance surgeons’ leadership of operating room teams (see List of Supplemental Material and Supplemental Fig 1).

## Material and Methods

### Research Setting/Design

We applied mixed methods to study cardiac surgery teams in an academic medical center performing more than 1,000 cardiac surgical procedures annually with outcomes meeting national benchmarks. Data were collected during two 4-month periods between September 2013 and April 2015. Each data collection period comprised: (1) a staff survey on team dynamics and surgeon leadership, (2) observations of surgeons’ interactions with team members during surgical procedures, and (3) semistructured interviews with team members to gain insights on contextual influences underlying observed interactions. Data from the 2 collection periods were combined after confirmation of little substantive change over time and subjected to cross-sectional analysis. The institutional review boards of participating centers approved this study. Supplemental Material-A presents detail regarding study methods and results.

### Sample

The study population included surgeons, scrub technicians/nurses, circulating nurses, physician assistants, perfusionists, anesthesiologists, and trainees (eg, surgical fellows, anesthesia residents). This included 7 surgeons and 116 nonsurgeons across the 2 data collection periods. Three nonsurgeons declined to participate and were excluded from the research.

In each data collection period, we surveyed all active surgeons and nonsurgeons in the sample, including 7 surgeons and 82 nonsurgeons in the first period and 5 surgeons and 105 nonsurgeons in the second period. We observed cases involving all 7 surgeons in the first data collection period and 4 of the 5 active surgeons in the second period (1 surgeon requested observations be discontinued). We conducted interviews with 34 surgical team members, including all surgeons and 1 to 3 team members from each discipline.

### Data and Data Collection

The survey (Supplemental Material-B) used 13 constructs of 1 to 3 items drawn from previously validated scales to measure surgical staff member perceptions and attitudes about themselves, their teams, and team dynamics. Nonsurgeons were also asked to evaluate the general performance of each surgeon as a team leader. Surveys were administered electronically and used a 7-point Likert scale.

We used an observation tool (Supplemental Material-C) to collect data about interactions between surgeons and nonsurgeons during individual surgical procedures. In addition to closed-ended items about case characteristics (date/time/location, type and difficulty, checklist use, level of surgeon participation), the tool largely comprised structured space to allow investigators to record all verbal and nonverbal interactions. Each surgeon was first observed by a team of 2 to 4 investigators to calibrate use of the tool, enhance its reliability, and acclimate surgical team members to our presence. After calibration, 1 research assistant (RA) observed each case. We pilot tested the tool in 23 cases (2 to 4 cases per surgeon) outside of formal data collection in the first period and again in 13 cases (2 to 5 cases per surgeon) outside of formal data collection in the second period. In total, the analytic sample included 22 cases (14 in the first period and 8 in the second period) comprising 110 observation hours. Average case duration was 5 hours, ranging from 1 to 9 hours.

Semistructured interviews asked participants to describe operating room team dynamics at their best and worst, frequency of and factors influencing such conditions, opportunities for improvement, perceived level of shared understanding among team members, and contextual influences underlying surgeon/team member interactions (Supplemental Material-D). In the second data collection period, we asked participants to comment on preliminary findings from the first period, resulting in modifications, as needed, of our initial interpretations. Interviews were conducted by 1 or 2 investigators, were confidential, lasted 15 to 60 minutes, and were digitally recorded and transcribed. Participation in all data collection was voluntary and without incentives.

### Analysis

Survey data from both collection periods were combined into a single analytic data set. For individuals who completed the survey twice, their responses were averaged and the mean taken as their score for each item. We calculated composite scores for each survey construct and generated distributions and descriptive statistics for all measures. To evaluate surgeon performance as perceived by surgical staff, we averaged responses provided by all nonsurgeons for the survey question on performance of the surgeon as a team leader.

During observation pilot testing, we performed qualitative coding to generate an initial set of behavior codes and definitions. After initial coding, we compared our empirically derived codes with previously published taxonomies for surgeon or surgical team member behaviors (Supplemental Material-E) 5, 6, 7, 8, 9, 10. Given little consensus among preexisting taxonomies and minimal overlap with our codes, we made only minor word choice changes. We then classified coded interactions for the 22 cases in our analytic sample into 33 behavior types. Each RA independently coded 5 transcripts to establish interrater reliability and coding consistency (kappa = 0.8; *p* < 0.0001) so that all remaining transcripts could be reviewed and coded by 1 RA.

We assigned a valence to each behavior type (positive, neutral, or negative) based on investigator assessment of the contribution of the behavior to more or less productive team dynamics. A neutral valence indicated ambiguity or that the behavior was contextually contingent. The 33 behavior types were then grouped into 7 distinct leadership functions derived through inductive and deductive processes informed but not determined by existing leadership literature. Using this leadership framework, we created profiles of surgeons’ leadership based on the frequency and proportion of each behavior and valence indicator. We did this for each surgeon, averaging across his or her cases, and for all surgeons combined.

To explore what might be considered “optimal” surgical leadership, we compared leadership profiles of the 2 surgeons with the highest-rated performance as team leader to the 2 surgeons with the lowest-rated performance. We calculated the percentage of each leadership function and valence and compared them using a χ^2^ test.

Interview data were transcribed and coded using Dedoose software to develop an understanding of the context in which the interactions occurred. We identified basic themes that captured elements of the operating and organizational environment that participants felt influenced team member interactions. We applied these iteratively to the interview data using principles of thematic analysis [11], revising, refining, and ultimately identifying 5 high-order global themes.

We tested the leadership framework by correlating leader profiles, including valence, with survey-based measures of surgeon leadership. We first explored the relationship between observed positive and negative forms of behavior with surgical staff members' perceptions of surgeons’ performance as team leaders. We then tested the relationship between each leadership function and this perceptual measure using Pearson correlation coefficients, treating *p* < 0.05 as significant.

## Results

Among the 123 individuals surveyed, 88 completed a survey in at least 1 of the 2 data collection periods. The response rate was 100% (7 of 7) for surgeons and 70% (81 of 116) for nonsurgeons. In the final analysis, we excluded 13 (11%) nonsurgeon respondents because of substantial missing/incomplete responses. Most of the remaining nonsurgeon respondents, representing a mix of disciplines, were women (53%), younger than 50 years (59%), worked at least 40 hours per week (75%), and had worked in the cardiac surgery unit for 5 years or less (54%) (Supplemental Material-A1). Although a majority of surgeons were also younger than 50 years (57%), in contrast to nonsurgeons, most surgeons were men (86%), worked at least 60 hours per week (100%), and had worked at the hospital more than 15 years (57%).

Consistent with research describing conditions in surgical units more generally [12], staff in this setting characterized teams as having relatively low levels of psychological safety, open communication, and perceived power (all <4.5 on a 7-point scale). Nonsurgeons perceived the openness of communication and of their own power and status in the operating room lower than did surgeons (*p* < 0.05) (Supplemental Material-A2).

The interview response rate was 83% (34 of 41), including 19 in the first data collection period (6 surgeons, 5 nonsurgeon leaders, and 8 nonsurgeon team members) and 15 in the second period (5 surgeons, 6 nonsurgeon leaders, and 4 nonsurgeon team members).

According to survey responses, surgical staff evaluated the performance of surgeons as team leaders as 5.4 of 7 (SD = 0.85), ranging from 4.2 for the lowest-rated surgeon to 6.2 for the highest-rated surgeon (Table 1). Different staff members’ evaluations of a given surgeon varied widely, although less so for higher-rated surgeons. For example, the surgeon with the lowest average rating received evaluations ranging from 1 to 7 (SD = 2.07). Evaluations for the highest-rated surgeon ranged from 3 to 7 (SD = 0.93).

**Table 1:** Perception of Surgeon Leadership and Surgeon Behavior by Function and Valence

Category	S1	S2	S3	S4	S5	S7^a^	S8	Surgeon Average
Perception of surgeon as team leader, average (SD)	6.22 (0.93)	4.22 (2.07)	5.17 (1.14)	6.02 (1.01)	5.81 (1.04)	4.75 (1.49)	5.91 (1.08)	5.40 (0.85)
No. of behaviors observed	218	317	201	417	218	129	426	275
Percent of behaviors by leader function								
Elucidator	24%	38%	17%	25%	14%	29%	20%	24%
Tone setter	17%	34%	15%	25%	14%	23%	13%	20%
Engagement facilitator	26%	6%	18%	15%	14%	9%	16%	15%
Delegator	10%	9%	14%	12%	22%	12%	25%	15%
Safe space maker	18%	9%	18%	14%	21%	13%	12%	15%
Conductor	4%	2%	11%	7%	15%	14%	10%	9%
Being human	0%	1%	6%	3%	0%	0%	4%	2%
Percent of behaviors by valence								
Positive	84%	49%	71%	72%	64%	43%	69%	66%
Neutral	15%	27%	22%	18%	28%	12%	29%	23%
Negative	1%	24%	7%	9%	8%	45%	2%	11%

a Numbering reflects exclusion from the study of 1 subspecialist surgeon. After initial observation, we realized that this surgeon did not interact sufficiently with other team members to warrant inclusion.

We coded 1,926 surgeon to nonsurgeon interactions (approximately 1 every 3.5 minutes) as 1 of 33 behaviors and grouped related forms of behavior into 7 distinct leadership functions: elucidator, tone setter, engagement facilitator, delegator, safe space maker, conductor, and being human (Supplemental Material-A3).

As elucidators, surgeons served as teachers, explaining their thought processes, instructing about specific maneuvers, updating the room about case progress or decisions, and providing public or private criticism of a constructive or destructive form. The elucidator function included 4 positive behaviors—teaching, constructive criticism, explanation, and relevance giving—and 2 negative behaviors—private criticism and negative criticism.

The tone setter function included 4 positive behaviors—constructive humor, compliments, reassurance, and encouragement; 2 negative behaviors—frustration and destructive humor; and 1 neutral behavior—conversation unrelated to the case.

As engagement facilitators, surgeons consulted team members for status updates, data, or their professional opinion; inquired with teammates about concerns; collaborated on shared tasks; helped with or supported another’s task; and expressed thanks and apologies to teammates. The engagement facilitator function included 6 positive behaviors—collaboration, consultation, helping/supporting, apology, thanks, and inquiry.

As delegators, surgeons sought help from their teammates by help-seeking (positive) or requesting (neutral) that they provide assistance with or complete tasks. Surgeons can be safe space makers by making the operating room safe for others to ask questions, express concerns, and share information.

The safe space maker function included 3 positive nonsurgeon-initiated interactions that reflect the sense of safety the surgeon creates in the operating room: nonsurgeon-initiated concern, questioning, and information sharing.

Surgeons as conductors guided their teams through series of surgical steps, returned the team’s focus to the task at hand when needed, anticipated and alleviated teammates’ concerns, closed loops to complete verbal exchanges about discrete tasks, and made requests in a way that required clarification for team members. The conductor function included 4 positive behaviors—returning the team members to focus, anticipating concerns, mapping steps, and closing loops for confirmation—and 1 negative behavior—the need for nonsurgeons to seek clarification.

Finally, surgeons led through showing their human side (“being human”) by self-questioning as 1 positive behavior, using idiosyncratic jargon as 1 negative behavior, and showing fatigue and musing as 2 neutral behaviors.

Interview findings identified elements of the operating environment that influenced team dynamics and the appropriateness and effect of leaders’ behaviors. These included (1) specifics of the surgical case, (2) personnel involved, (3) group’s collective perception of the importance of social as well as technical competence, (4) more enduring organizational factors such as equipment management and staffing practices, and (5) cultural/historical factors like reputation of the hospital, perceived priorities, and mental models of teamwork.

### Variation in Surgical Leadership

Frequency and proportion of specific behaviors varied. Of 275 behaviors observed, 66% were positive and 11% were negative. The most frequent surgeon behaviors reflected their role as elucidators (24% of interactions) and tone setters (20%).

For individual surgeons, there was wide variation in the valence of behaviors and leader functions enacted. Positive behaviors accounted for 84% of interactions for surgeon 1, but only 43% for surgeon 7. For these same surgeons, negative behaviors composed 1% and 45% of interactions, respectively. Among leader functions, the percentage of behaviors contributing to surgeons’ elucidator function varied most (14%–38%). Leading by demonstrating one’s human side varied less (0% for surgeons 1, 5, and 7 to 6% for surgeon 3). Some surgeons expressed certain leader functions preferentially, whereas others were more balanced in their exercise of different leadership functions. Surgeon 3 directed a maximum of 18% and a minimum of 6% of behaviors to each of the leader functions. In contrast, surgeon 7 devoted almost a third of his behaviors to elucidating (29%), none to being human (0%), and few to facilitating others’ engagement (9%).

**Fig 1:**
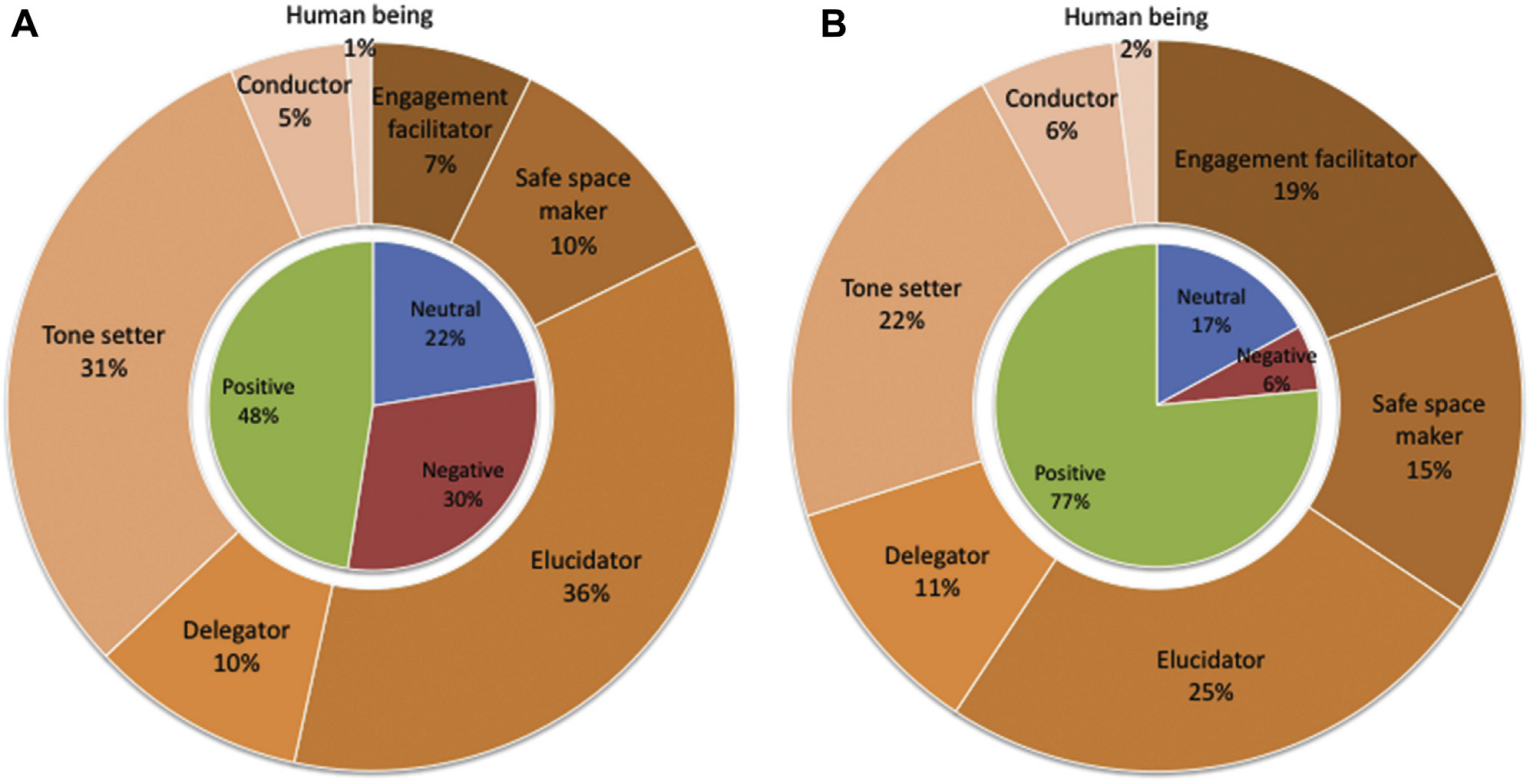
Leadership profiles. (A) Average of 2 lowest performers and (B) average of 2 highest performers in terms of perceived team leadership.

When comparing the combined profiles for the 2 surgeons perceived as highest and the 2 perceived as lowest in team leadership, some similarities and clear differences emerged (Fig 1). The greatest difference between the highest- and lowest-ranked surgeons was in facilitating engagement (19% versus 7%). Although surgeon as elucidator was the most common leader function for both high- and low-ranked surgeons, elucidating comprised a higher proportion of behaviors for the lowest-ranked compared with the highest-ranked surgeons (36% versus 25%) as did tone setting (31% versus 22%). The lowest ranked surgeons enacted all other leader functions less frequently than did the highest-ranked surgeon.

For leadership functions composed of both positive and negative behaviors, the highest-ranked surgeons engaged in more positive behaviors than the did lowest-ranked surgeons (Supplemental Material-A4). The highest-ranked surgeons elucidated through positive behaviors 91% of the time, whereas the lowest-ranked surgeons elucidated in positive ways just 53% of the time. Similarly, the highest-ranked surgeons enacted tone setting through positive behaviors 61% of the time compared with 19% of the time for the lowest-ranked surgeons. Overall, the proportion of positive behaviors for the highest-ranked surgeons was 77% compared with 48% for the lowest-ranked surgeons. The difference between the distributions of higher- and lower-ranked surgeons’ behaviors by leadership functions and valence are both significantly different (*p* < 0.0001).

### Comparison of Leader Profiles With Staff Perceptions of Surgeon Leadership

We examined the relationship between observation and survey data to determine the consequences of leader behavior on perceptions of surgeon leadership (Supplemental Material-A5). Positive behaviors were strongly positively correlated (*r* = 0.85; *p* = 0.02), and negative behaviors were negatively correlated (*r* = –0.75; *p* = 0.05). Within leadership functions, facilitating engagement related most positively (*r* = 0.80; *p* = 0.03) and negative forms of elucidating (negative and private criticism) (*r* = –0.81; *p* = 0.03) and tone setting (frustration and destructive humor) (*r* = –0.68; *p* = 0.09) related negatively to team member perceptions of surgeon leadership. Interview findings suggested that staff members understood that criticism was a long-standing teaching tool intended to instruct rather than demoralize; however, personal attacks interfered with staff’s ability to learn and willingness to speak up. In contrast, staff members described enthusiasm for opportunities to engage and learn.

## Comment

This study sought to identify leadership behaviors that support surgical teamwork. We developed a leadership framework, which suggests that leader effectiveness derives from various forms of interactive behavior in the operating room; these behaviors may have positive, neutral, or negative valence, and the appropriateness and effect of leaders’ behaviors are shaped by contextual factors. We identified 7 leadership functions and related behaviors that impact perceptions of surgeons’ leadership. The ways surgeons interacted with team members varied greatly. Our analyses suggest that not only the functions performed but also the valence of behaviors through which surgeons as leaders enact those functions, affect team dynamics. Surgeons may benefit from deeper understanding of the impact of these behaviors through training in interpersonal leadership in addition to skill-based training.

Surgeons’ behaviors strongly influenced how operating room staff perceived their leadership. Negative criticism—however well intended—had a particularly harmful effect on perceptions. This finding is consistent with previous research showing that negative feedback and exposure to rudeness negatively impact individual self-concept, team performance, staff burnout, and turnover [13]. In contrast, engaging and willingness to seek help from team members related particularly positively to perceptions of surgeons’ leadership. Interviews confirmed that what nonsurgeon team members sought from surgeon leaders was collaboration, psychological safety, and opportunities to learn.

Perceptions of surgeons’ performance as leaders also varied greatly among team members. For some surgeons, leadership ratings clustered at high levels; for other surgeons, ratings varied. This indicates a lack of consensus about the appropriateness of particular forms of behavior or the willingness to overlook negative behavior, particularly when interpersonal relationships were strong or when other contextual contingencies were present. This suggests that assigning appropriateness to surgeons’ behaviors requires attention to context. Team members claimed to understand that there are times for leaders to be more dominant and controlling (eg, during a difficult case, under time pressure). However, we also observed different responses to contextual contingencies: eg, surgeons’ reactions to lack of case preparation resulting from late assignment to a case varied widely, suggesting room for surgeon discretion even in the presence of contingencies. Importantly, when negative behaviors are not warranted or personal, team members perceive them as destructive to the ability of teams to interact effectively.

Our taxonomy complements several high-quality coding schemes for surgeon and surgical team member behavior 5, 6, 7, 8, 9, 10. Existing taxonomies characterize behavior differently, focusing on team activities 5, 6, surgeons’ nontechnical skills 7, 8, or teaching behaviors 9, 10. Most existing taxonomies are derived from classification schemes suggested by other industries, including aviation 5, 6, leadership literature [7], or input from surgeons or surgical team members 7, 8, 10. Only 1 [9], which focused exclusively on surgeons’ teaching behaviors, relied on direct observation. In contrast to other qualitative forms of data collection, observation allows identification of norms that may be taken for granted and of actual practice, not mediated by participants’ perceptions of what may be relevant or interesting to investigators [14]. An observation-based taxonomy that focuses on surgeons’ leadership, not just teaching, provides the potential for deeper understanding of what enhances and undermines teamwork and team performance.

Our framework focuses on leadership functions and behaviors. Our taxonomy shares elements with previous classification schemes but also identifies behaviors found infrequently elsewhere. Although several behavior types had a low frequency in our sample (ie, help-seeking, clarification, self-questioning, jargon), each was present and clearly distinct from the other forms of behavior observed. Earlier taxonomies often focus only on leadership functions (eg, briefing, vigilance, awareness) rather than on ways of enacting those functions. The greater specificity offered by our framework adds value by offering specific actionable targets—both productive and unproductive behaviors—for improving team performance.

Previous studies have not related nontechnical behaviors to the quality of surgeons’ leadership. Rather, previous taxonomies demonstrate observer accuracy and agreement when applied to observed or videotaped surgical cases 6, 7, 15, 16, variation in behaviors among teams and surgeons 17, 18, improvement with training 6, 19, relationship of nontechnical behaviors with lower errors [6] and odds of complications and death [5], and improvement in other indicators 15, 19. Thus ours is the first validated overarching conceptual framework that demonstrates how surgeon behaviors influence perceptions of surgeons’ leadership and surgical teamwork. The way team members regard their surgeons’ leadership suggests how well the team will function and thus provides a leading indicator of team performance and patient safety.

### Limitations

Study limitations should be considered. First, this was a small sample size with limited statistical power. This may have limited the range of leadership behaviors and functions we observed. Thus we cannot claim our taxonomy is comprehensive. Nevertheless, our leadership framework identified granular, and thus actionable, leadership functions. Second, we did not relate perceived quality of team leadership to clinical outcomes. Previous research has established this association [4]. Third, substantial turnover in personnel, including trainees and staff surgeons, complicated our ability to combine survey data across data collection periods and did not allow us to complete data collection for all the original surgeons. Fourth, nonparticipation could have resulted in selection bias and reduced observed differences if reluctance to participate resulted from self-knowledge of negative behaviors. Fifth, observation data may have been influenced by observer bias (Hawthorne effect). However, extended acquaintance periods acclimated surgical team members to our presence.

### Conclusions

Our findings suggest concrete behavioral strategies that surgeons can use to improve team performance, in particular by reducing negative forms of criticism and increasing engagement of team members in perioperative tasks. Further research should aim to test, in different settings, theories that our leadership framework introduced and illuminate more specific relationships between types of contextual factors, team member characteristics, and surgeons’ behaviors to establish more direct links between nontechnical competencies and surgical outcomes. The diagnostic profiling approach we have developed may be adopted to collect and display individualized communication and leadership data for future research and improvement. Likewise, this approach could be used by graduate medical education programs to teach trainees to modify their behavioral repertoire and reduce negative behaviors.

**Author Interview:** The Author Interview can be viewed in the online version of this article [http://dx.doi.org/10.1016/j.athoracsur.2017.01.021] on http://www.annalsthoracicsurgery.org.

## Discussion

**DR OURANIA PREVENTZA** (Houston, TX): That was an outstanding paper. I would like to congratulate you as well as congratulate Dr Sundt for this initiative. I do have 2 questions for you. Were the cases that you observed emergent and elective, or only elective cases?

**MS STONE:** Elective cases.

**DR PREVENTZA:** And then my other question is, what were the qualifications of the observers? You said there were different observers in the operating room observing Dr Sundt to do a case. What do you think the qualifications should be, and who actually will be responsible to tell us the results?

**MS STONE:** One of the benefits of this specific research team was that we came from many different disciplines, so we had a professor from the school of public health with experience studying leadership in various health care settings, we had someone from the business school who studies organizational psychology and teams in business settings. Dr Sundt and Dr Wright advised our observation team as well.

**DR PREVENTZA:** I think this is very important, because let's say you do a very high-risk elective case. The level of stress is completely different for a hernia repair versus a total arch reconstruction. For example, let’s say there is an observer from outside of the medical environment (not a health care practitioner) that is an expert on leadership but has no idea about the case that the cardiothoracic surgeon is about to perform and what risks it really entails. Is this expert the right person to report and judge? I would like your thoughts on that.

**DR SUNDT**: Sorry to jump in. We weren't making value judgments about people. I was a subject too, so I was blinded to the data collection and to the analysis until it was all over. I will also mention I was not on the high end of that spectrum either, but the observations were of behaviors, so they were behavioral scientists coding behavior specifically.

**DR PREVENTZA:** Excellent paper. Great presentation.

**DR ELIZABETH STEPHENS** (New York, NY): I just want to echo what a phenomenal study and a great presentation you gave. You should be congratulated on this.

My specific question is whether you looked at how behaviors may have responded to adversity. When I think of surgeon leaders that I admire, largely it is how they respond to something going wrong in the OR and how they rally the team in the context of adversity. Did you have an opportunity to look at that?

**MS STONE:** Yes, that is a great question. We noticed a range of responses to adversity. On 1 extreme, certain surgeons would react to adversity by increasing the amount of information they shared; other surgeons on the other extreme would react by only sharing information on a need to know basis or when it was absolutely necessary. Dr Sundt?

**DR SUNDT:** A really critical difference observed was how much information was shared by the surgeons with the team.

**DR AMANDA EILERS** (San Antonio, TX): I wanted to also commend you on your work, especially as a premedical student. I was in your shoes when I had my first mentor, and I was really taken by his positivity and how he carried himself as a leader in the OR and outside of the OR, so I really hope that you use this in your journey not only as a premedical student but also in your journey to hopefully become a cardiothoracic surgeon.

I had 2 comments. I think all of us in the room can pick out from an eyeball test who is a good patient for a certain operation, and I think as a trainee we learn throughout our career who we want to emulate as a leader in the operating room and in a clinic setting, so I think this really is exciting data and I look forward to what you are able to study down the line. Thank you so much for your work. I appreciate it.

**MS STONE:** Thank you for that comment.

**DR FAISAL BAKAEEN** (Cleveland, OH): A very provocative and interesting study, and congratulations on an excellent presentation. We are evaluating surgeon S1 through maybe S6. Everything else could be a variable. Did they operate with the same nurses? Did they do the same complexity of cases, and did we adjust for generational issues? For example, was it a young nurse versus an older nurse, a young PA versus an old PA? I think these kind of things are probably your next step to look at it from a more granular level, but what would be really interesting to see is if that leadership performance of the surgeon correlated with efficiency outcomes such as OR turnaround time or duration of a case, and whether it actually impacted risk-adjusted outcomes of the patients. Thank you very much.

**MS STONE:** Thank you for that comment. I think that is a key area for future research for us. In this study we had a relatively small sample size, and some of the outcomes we were considering occurred relatively infrequently, so we didn't necessarily have the statistical power to look at those relationships, but I agree that would be an essential next step.

**DR DIPESH SHAH** (Pittsburgh, PA): Excellent presentation and a very interesting topic. Dr Sundt clarified that the subject was blinded and your slide stated Hawthorne effect as 1 of the limitations. I think the Hawthorne effect is biggest when it comes to the human behavior when they are being watched and especially when they know they are being watched, so I wanted you to clarify a little bit about that. How was the Hawthorne effect a limitation if the subject was blinded.

**MS STONE:** We knew the Hawthorne effect could be a potential limitation for the study, and we attempted to mitigate that initially through conducting a series of pilot observations. We conducted about 30 different pilot observations to acclimate the team to our presence and also to trial out our observation tool to make sure it was collecting the data we needed.

Also I need to mention that this study was done in an academic medical center, and the teams were pretty used to presence in the operating room, whether from students or other researchers, so our presence there wasn't abnormal, and then, finally, we stood in the back of the room and kept a very low profile and blinded the subjects to what we were collecting.

Also 1 last point is we still saw “bad behavior” occur despite our presence, so, yes, the teams may have been putting on a show in the beginning, but they certainly acclimated to our presence after a while.

**DR WILLIAM A. BAUMGARTNER** (Baltimore, MD): Juliana, it was a terrific presentation, amazing study, and I commend Thor for having the courage to do this kind of study.

Your conclusion is that positive leadership qualities can be taught. What are the next steps? I gather these data were shared with the individual surgeons, and now what is the educational process? To me, that is the most important next step following your analysis.

**MS STONE:** That is a good question; so, as mentioned, we did show these profiles to the surgeons in our sample. The first step in improving leadership is just to understand how you lead, and a lot of the surgeons were surprised to see that they were dominant in certain areas of leadership over others. They also were surprised to see where they fell in relation to their fellow surgeons in terms of their team members' perceptions of their leadership. That was informative.

And then the second step was having the ability to indicate to surgeons which forms of their leadership were working better for their teams than others. We were able to do that through the study through those correlations.

And then the third step after seeing it and knowing which areas are working better than others is to try to make some changes, and we saw this done well when surgeons could start small and create habits, so we know that the more surgeons can facilitate the engagement of other teammates, the higher their teammates' perceptions of their leadership is, but you can see that there is a set of behaviors that allow surgeons to enact the engagement facilitator function. So 1 of the surgeons would focus on 1 of these behaviors, say consultation, think about it right before walking into the operating room, and before he walks to the table would go to each one of his team members and check in on what was going on from their perspective in the case. That became a habit for him—1 first step along the path of improvement.

## Supplementary Data

List of Supplemental Material

Supplemental Material-E

Author Interview
