# Supplementary material for: Effective Leadership of Surgical Teams: A Mixed Methods Study of Surgeon Behaviors and Functions
Source: Ann Thorac Surg. 2017 Aug;104(2):530–7. doi: 10.1016/j.athoracsur.2017.01.021 (PMC5527126; doi:10.1016/j.athoracsur.2017.01.021)
Supplement: List of Supplemental Material [file mmc1.docx]

**LIST OF SUPPLEMENTAL MATERIAL**

Supplemental material-A: Detailed methods

Supplemental material-A1: Sample Characteristics for Surveys, Interviews, and Cases Observed

Supplemental material-A2: Survey Questions 1-16 Overall and by Role (Surgeon, Non-Surgeon)- Pre and Post Surveys Combined

Supplemental material-A3: Behavior types by leadership function with a description and examples

Supplemental material-A4: Comparison of leadership function and behavior valence for the two highest and two lowest ranked surgeons

Supplemental material-A5: Correlation between leadership function (observations) and non-surgeon perception of surgeon as team leader (survey)

Supplemental material-B: Cardiac surgery team dynamics non-surgeon team member survey

Supplemental material-C: Cardiac surgery observation tool

Supplemental material-D: Interview guides

Supplemental material-E: Comparison of leader behavior taxonomy to prior tools
