# Supplementary material for: Effective Leadership of Surgical Teams: A Mixed Methods Study of Surgeon Behaviors and Functions
Source: Ann Thorac Surg. 2017 Aug;104(2):530–7. doi: 10.1016/j.athoracsur.2017.01.021 (PMC5527126; doi:10.1016/j.athoracsur.2017.01.021)
Supplement: Supplemental Material-A [file mmc2.docx]

**SUPPLEMENTAL MATERIAL-A: DETAILED METHODS**

Effective leadership of surgical teams: A mixed methods study of surgeon behaviors and functions

As the research upon which our study relies entails multiple sources of data and multiple analytical strategies, the presentation of methods in the main manuscript are summarized in order to respect space constraints. The purpose of this supplement is to provide more detailed information about the methods applied in this research and to present supplemental results. To facilitate its interpretation, we have organized the supplemental material in the same order in which the methods are presented in the main manuscript.

### *Research setting*

The hospital in which we conducted this research is a major metropolitan academic medical center in the Northeast that ranks among the top hospitals in the country for teaching, research, and clinical care. The cardiac surgery division has outcomes that consistently meet or exceed those of similar institutions as reported by the Society of Thoracic Surgeons [1].

The cardiac surgery division performs over 1,000 surgical cases per year. Procedures performed include coronary artery bypass grafting, valve repair and replacement, aortic surgery and heart replacement therapy including ventricular assist device and transplant. Team composition is conventional, but as a teaching hospital, also includes trainees (e.g. surgical fellows, anesthesia residents) who actively participate. Non-surgeon team members rotate with every case, and sometimes within a case.

### *Research design*

We studied cardiac surgical teams using mixed methods. We defined surgical teams as the multidisciplinary group of individuals in the operating room contributing to surgical care of the patient during a given case. Data collection occurred between September 2013 and April 2015 over two four-month periods, separated by a pause during which we provided preliminary feedback to study participants. The presence of a pause allowed investigators to take stock of saturation levels and adapt data collection methods.

Each data collection period comprised a staff survey, observations of surgical procedures, and interviews with surgical staff and leaders from each surgical discipline. We surveyed cardiac surgical personnel about team dynamics in their operating rooms. In addition, we asked non-surgeon cardiac staff to evaluate surgeons’ performance as team leaders. We observed surgeon-team member interactions during cardiac surgical cases in order to understand what leadership functions surgeons fulfill in the operating room and what surgeon behaviors enact those functions. We conducted semi-structured interviews with cardiac division members to deepen our understanding of the contextual influences underlying surgeon-team member interactions.

After confirming little substantive change overall in survey and observation results between the initial and subsequent data collection periods, we combined the data over time and performed cross-sectional analyses. We drew on all three data sources to develop a conceptual framework of surgeons’ leadership functions, the behaviors that enact each function, and the contextual factors that influence surgeon-team member interactions. We validated the conceptual model by comparing surgeons’ leadership behaviors and functions to staff perceptions of each surgeon’s leadership. The Institutional Review Boards of the authors approved this study.

### *Sample*

The study population for all data collection methods comprised personnel from each professional discipline (surgeons, anesthesiologists, anesthesia nurses, circulating nurses, scrub nurses, surgical technicians, perfusionists, physician assistants, and surgical trainees, i.e., residents and fellows) within the cardiac surgery division of our study hospital. To identify potential participants, we obtained names and contact information for active members of each professional group from the leaders of each discipline prior to each data collection period. Cumulatively, our population included initially eight surgeons and 119 non-surgeons. We excluded from analyses one surgeon, who specialized in retrieving donor organs for transplant patients. After initially observing this surgeon, we realized he did not interact sufficiently with other team members to be included in the study. Also, of the 119 non-surgeons, three declined consent for participation in the study, one by opting out of the survey, one through a verbal request, and another through a written request. These individuals were excluded from all components of the research. Thus, our final sample included seven surgeons and 116 non-surgeons.

Survey. We sent the survey to all surgeons and non-surgical staff in the sample. In the first data collection period, we surveyed seven surgeons and 82 non-surgeon team members. For the second data collection period, we surveyed five surgeons—two had left the division—and 105 surgical team members—11 staff had left the division from the original sample and 34 staff members had joined the division according to discipline leaders. We performed a two-tailed, paired t-test to compare the distributions of nurses, anesthesiologists, trainees, and others that responded to the surveys in the first and second data collection periods. A significant difference by type of personnel would raise concern for potential bias due to systematic differences in perceptions of surgeon leadership by discipline. However, we found that the distributions of respondent by discipline in the first and second data collection periods did not differ significantly (p=0.50). We added this information to the technical appendix.

Observation. We observed seven surgeons. In the initial data collection period, this included six surgeons who were present in the division at the commencement of data and one surgeon who joined the division after we commenced initial data collection. We conducted initial observations for this surgeon before proceeding to the second round of data collection. Before the second data collection period, two surgeons left the division and one requested to discontinue observations. Thus, in the second data collection period, we observed a total of four surgeons. We did not track the specific team members observed during surgical cases.

Interviews. In total, we conducted interviews with 34 surgical team members. Interviewees included the seven surgeons in our sample, as well as one leader and one to three team members recommended by the leader from each surgical discipline: anesthesiologists, nurses, perfusionists, physician assistants, and surgical trainees). In the first data collection period, we invited 24 individuals to interview (including seven surgeons, five non-surgeon leaders, and 12 non-surgeon team members). In the second data collection period, we invited 17 individuals to interview (six surgeons, five non-surgeon leaders, and six non-surgeon team members).

### *Data*

Survey. We developed a survey (Supplemental Material-B) to measure surgical staff member perceptions and attitudes about themselves, the team, and team dynamics in their operating rooms. Most of the survey was designed to provide descriptive information about the research setting. Survey items asked each surgeon and non-surgeon to self-report about their personality using the “big 5” personality traits, and about their perceptions of surgical team dynamics, using 13 constructs, including self-efficacy, social worth, job satisfaction, burnout/emotional exhaustion, power, status, identification, psychological safety, open communication, coworker relationship quality, individual learning, team learning, and team confidence. Constructs of one to three items each were drawn from previously validated survey scales

[2-16]. In some cases investigators selected subsets of items from particularly long scales or modified items slightly to enhance applicability to the cardiac surgical context.

We included additional items directed to non-surgeons only, in order to assess non-surgical staff members’ impressions of the cardiac surgeons with whom they work. Survey items asked respondents to evaluate the general performance of each surgeon as a team leader. In addition, specific items asked staff to evaluate the surgeons’ openness to new ideas; receptivity to suggestions; interest in others’ perspectives; desire to have everyone obey him/her; whether/how much the surgeon makes the respondent feel pressure; and whether/how much the surgeon scolds other team members. In the second survey, we added additional exploratory items requested by the surgeons regarding team members’ enthusiasm for assignment to the surgeon’s operating rooms and engagement in the surgeon’s cases. Since these data are not available for all survey respondents, we exclude them from our analysis. All survey items used a 7-point Likert scale, where “1” meant strongly disagree and “7” meant strongly agree.

Observation tool. We developed an observation tool (Supplemental Material-C) that enabled us to collect data about interactions between surgeons and other members of the surgical team during a surgical procedure. The multi-page instrument was pilot tested in cardiac cases before its use for official data collection and, once finalized, it was used for all observations.

Closed ended items collected information about case characteristics including the date, time, duration, location, type and difficulty of the procedure, whether the team used a surgical checklist before anesthesia, before incision, before perfusion, and before patient left the operating room, whether the surgeon was present to perform them, and whether an overhead or headlight camera was used to display a live video of the surgical field on a monitor fixed on the wall of the operating room. Closed-ended items, intended for completion after the procedure, documented deviation from the surgical plan or from regular behavior in the operating room.

Most of the observation tool was devoted to structured blank space intended to allow investigators to record verbal and nonverbal interactions between the surgeon and another member of the surgical team (i.e., one column was used to record interactions between the surgeon and the anesthesiologist, another for the surgeon and the perfusionist, etc.). This section of the tool could be expanded as needed by adding pages devoted entirely to recording these interactions. The tool did not capture information about interactions between dyads not involving surgeons. Each data element consisted of a discrete exchange in the form of a word, phrase, dialogue or physical overture. In addition, open-ended items to be completed after the procedure allowed an observer to record her impression of overall team dynamics: the degree of rapport and collaboration practiced by the surgeon with his/her team, whether the room felt relaxed or tense, and any strengths, weaknesses, or concerns of note that day.

Interview protocol. Interviews sought to deepen our understanding of contextual influences underlying surgeon-team member interactions. We developed semi-structured interview protocols to guide conversations with staff members at the outset of the research and at its conclusion.

At the initial interviews, we asked participants to describe operating room team dynamics at their best and worst and how frequently the participant experienced these conditions. We also asked about factors influencing team dynamics and how they could be improved. At concluding interviews, we asked participants to comment on preliminary findings, which we shared with each disciplinary group and individually, in the case of surgeons. In addition, we asked who they considered to be part of their team, the extent to which they felt other team members understood their role, and their views on the changes needed to achieve their vision of ideal team dynamics. Interview guides available in Supplemental Material-D.

#### Data collection

Survey. We administered the staff survey twice as part of each data collection period, in December 2013 and February 2015. We did so electronically, using Qualtrics, a university-sponsored electronic survey tool. For each survey administration, we sent email reminders approximately weekly. We also encouraged role leaders to remind staff to complete the survey at staff meetings or via email. With each administration, the survey remained open for completion for about two months. Survey participation was voluntary, and subjects could decline to participate by not responding to the survey. We provided no financial incentive for participation in the survey.

Observations. Investigators observed each surgeon over multiple days. Initially, we pilot-tested the tool in observations of each surgeon over a minimum of two days (1-2 cases per day). For at least one of the cases for each surgeon, we observed in teams of two to four investigators in order to develop a shared understanding of each surgeon’s patterns of interaction. Observation teams included one of two pre-medical research assistants, who would perform the formal data collection, and at least one of the senior investigators. This pilot-testing allowed investigators to calibrate use of the observation instrument in order to enhance its reliability. These observations also acclimated surgical team members (who as teaching hospital staff were already quite accustomed to observers) to our presence. Before each observation, and as new staff members joined the surgical team, we consented personnel who had not previously returned a consent form. We also answered questions about the purpose of our study, explaining that our objective was to observe the team in order to provide feedback about team dynamics and reminding them that data collected would be de-identified and used in aggregate form.

After acclimatization, one of the pre-medical research assistants observed each surgeon for two additional cases on different days for purposes of data collection, during the first four-month data collection period and again during the second four-month data collection period. During the second data collection period, a senior investigator also joined for one case per surgeon. In total, we pilot-tested the tool in 23 cases (average of 3.3 cases per surgeon, ranging from 2 to XX) prior to use for formal data collection in the first period. We conducted observations in 13 cases (average of 3.3 cases per surgeon, ranging from 2 to XX) prior to formal data collection in the second period to renew team member comfort with the presence of researchers.

Given the arrival and departure of surgeons from the division over the course of the study period and the request from one surgeon to discontinue observations after two cases, the total number of cases in the analytical sample was 22, comprising approximately 110 observation hours. This included 14 observed cases (two each) across seven surgeons (excluding the donor organ specialist but including the new surgeon) during the first data collection, and eight observed cases (two each) across four surgeons (excluding the donor organ specialist, two who left the division, and one who chose to discontinue observations) during the second data collection period.

Observers dressed in scrubs and stood in the back of the operating room often alongside students or other unrelated observers, approximately 10 feet from the operating table. This allowed observers to hear and see team member interactions with reasonable accuracy while staying out of the way of the team and keeping a relatively low profile. Observers recorded observations in writing, using the observation tool. During slower periods, observers could ask questions of the circulating nurse or perfusionist in order to better understand the happenings in the room. Upon completion of the case, investigators conferred with a non-surgeon team member to determine whether there had been any deviation from the surgical plan or from regular behavior in the operating room.

Interviews. One or two investigators conducted on site interviews, in a private room or office in the participant’s work area. Interviews were voluntary and confidential. They lasted from 15 to 60 minutes and were digitally recorded and transcribed.

### *Analysis*

Survey. First, we calculated response rates for the surveys from both data collection periods. Then, we combined data from the surveys obtained in both data collection periods to create our analytical dataset. Specifically, for individuals who completed the survey twice, we averaged their response for each item and used the mean response. For individuals who completed the survey once, we used their single response to represent their score for each item. We then calculated composite scores for each survey construct. We then generated scores for each survey construct by averaging relevant item scores for each individual. We generated distributions and descriptive statistics for all survey measures, first overall and then comparing surgeons to non-surgeons. We did not analyze survey data at a more granular level, e.g., by professional discipline, due to staff concerns about confidentiality.

Our primary use of the survey was to create a measure of surgeon performance as perceived by surgical staff. To do this, we averaged the responses provided by all non-surgeons for each surgeon. Given high levels of correlation between the measure of general performance of the surgeon as a team leader and the items measuring specific aspects of leadership (*r* = 0.90 to 0.97), we elected to use the general performance measure as dependent variable. Missing data for this variable was minimal (4% for the first data collection period and 2% for the second data collection period). We therefore simply ignored this missing data.

Observations. Observers transcribed the contents from the paper-based tool into an electronic file. While still in the pilot-testing phase of observations, we performed qualitative coding of observation data to generate an initial set of behavior codes. We compared our empirically-derived codes with previously published taxonomies for surgeon or surgical team member behaviors (see Supplemental Material-E for comparison) [17-20]. Given little consensus among preexisting taxonomies and minimal overlap of our codes with any of them, we made only minor word choice changes based on this comparison. Thus we used a combination of inductive and deductive coding to generate initial codes.

Through discussions among research team members, investigators continued using a constant comparative method

[21] to identify emerging themes, comparing them across surgeons’ cases and to extant literature, and refining the set of interaction type codes throughout both observation periods. At the conclusion of data collection, the set of codes totaled 33 behavior types.

For the observations that were part of the formal analytical sample, two research assistants (one of whom who was also an observer) assigned codes representing the list of behavior types to data elements from the observations. They both independently coded five transcripts, and we evaluated inter-rater reliability using a Kappa score to establish coding consistency. The two independent raters demonstrated near perfect inter-rater reliability in coding the observed operating room interactions (Kappa=0.8, p<0.0001). The research assistant who had not performed observations coded the remaining transcripts from the first wave of observations; the research assistant who had performed observations coded the second wave. She also reviewed the transcripts from the first wave and identified codes she felt were misattributed. Such discrepancies were resolved through group discussion with the full investigator team.

Once coding was complete, we returned to our primary research questions, ‘what are the leadership functions of surgeons in the operating room and what surgeon-team member interactions enact those functions?’ We developed a leadership framework to address these questions by organizing related interaction codes into topical groups. Drawing on existing leadership literature, we named the leadership function to which those interactions contributed. Ultimately, our 33 behavior types grouped into seven leadership functions.

We also assigned an indicator of valence to each behavior code (positive, neutral, or negative) based on observers’ assessment of the contribution of the behavior to more or less productive team dynamics. We designated behaviors as positive or negative based on observers’ consideration of the body language of those involved and the reaction of others in the room to the behavior and on theoretical and empirical literature that describes what makes for safe and productive team dynamics. A neutral valence indicated that the valence was ambiguous (i.e., not clearly positive or negative) or that the behavior was contingent, i.e., could be positive or negative depending on the situation. We then grouped the 33 behavior types into seven distinct leadership functions. As before, these higher-order conceptual categories were derived through a combination of inductive and deductive processes, being informed but not determined by conceptualizations from the existing leadership literature

[21].

Next, using this leadership framework we created profiles of surgeons’ leadership to understand the extent to which leadership varied in the operating rooms we had observed. Using observation data from both data collection periods, we calculated the frequency and proportion of each behavior type for each surgeon for each case. We then averaged these frequencies and proportions for each surgeon across all their cases to create surgeon-specific profiles and for all surgeons across all cases to calculate an average surgeon profile. For each surgeon and for the group of surgeons, we also calculated the average proportion of positive, neutral, and negative behavior types and the average proportion for each leadership function across all applicable cases.

To explore what might be considered “optimal” surgical leadership, we compared the average type and valence of behaviors of the two surgeons with the highest rated performance as team leader to the two surgeons with the lowest rated performance as team leader [22]. Specifically, we calculated the percentage of each leadership function and valence for the two highest and for the two lowest performing surgeons and compared their distributions using a chi-squared test.

Interviews. Investigators transcribed and coded interviews, using Dedoose software, to develop an understanding of the context in which surgeon-team member interactions took place. Analysis proceeded iteratively using the principles of thematic analysis [23]. We identified basic themes that captured elements of the operating environment (within and beyond the operating room), which participants felt influenced the nature of team member interactions. These themes were iteratively applied to the interview data, revised, refined and ultimately grouped into five high-order global themes. Interviews from the second data collection period informed modifications as needed of our initial interpretations of results from the initial data collection period.

Correlations. We tested the framework by performing a correlational analysis between leader profiles, including valence, with survey-based measures of surgeon leadership. We first explored the relationship between positive and negative forms of behaving (from observation data) with surgical staff member perceptions of surgeons’ general performance as team leaders (based on survey measures). We then tested the relationship between each leadership function and this perceptual measure. We assessed the correlations using Pearson correlation coefficients and considered p-values <0.05 to be significant.

**REFERENCES**

[1] Society of Thoracic Surgeons. Society of Thoracic Surgeons Public Reporting Online. Http://WwwStsorg/Quality-Research-Patient-Safety/Sts-Public-Reporting-Online 2015;Accessed March 1, 2016.

[2] Gosling SD, Rentfrow PJ, Swann WB Jr. A very brief measure of the Big-Five personality domains. Journal of Research in Personality 2003;37:504–28. doi:10.1016/S0092-6566(03)00046-1.

[3] Bandura A. Multidimensional scales of perceived self-efficacy. Stanford, CA: Stanford University; 1990.

[4] Keyes CLM. Social Well-Being. Social Psychological Quarterly 1998;61:121–40.

[5] Grant AM. The significance of task significance: Job performance effects, relational mechanisms, and boundary conditions. J Appl Psychol 2008;93:108–24. doi:10.1037/0021-9010.93.1.108.

[6] Quinn RP, Shepard LJ, Administration USES, University of Michigan. Survey Research Center. The 1972-73 quality of employment survey. 1974.

[7] Maslach C, Jackson SE. Maslach: Maslach burnout inventory. Palo Alto, CA: Consulting Psychologists Press, Inc; 1981.

[8] Anderson C, John OP, Keltner D. The personal sense of power. J Pers 2012;80:313–44. doi:10.1111/j.1467-6494.2011.00734.x.

[9] Tost LP, Gino F, Larrick RP. When Power Makes Others Speechless: The Negative Impact of Leader Power on Team Performance. vol. 56. Academy of Management Journal; 2013.

[10] Fragale AR, Rosen B, Xu C, Merideth I. The higher they are, the harder they fall: The effects of wrongdoer status on observer punishment recommendations and intentionality attributions. Organizational Behavior and … 2009.

[11] Henry KB, Arrow H, Carini B. A Tripartite Model of Group Identification: Theory and Measurement. Small Group Research 1999;30:558–81.

[12] Edmondson AC. Psychological Safety and Learning Behavior in Work Teams. Administrative Science Quarterly 1999;44:350–83.

[13] Sheldon KM, Bettencourt BA. Psychological need‐satisfaction and subjective well‐being within social groups. Br J Soc Psychol 2002;41:25–38. doi:10.1348/014466602165036.

[14] Shoss MK, Witt LA, Vera D. When does adaptive performance lead to higher task performance? J Organiz Behav 2012;33:910–24. doi:10.1002/job.780.

[15] Grant AM, Gino F, Hofmann DA. Reversing the extraverted leadership advantage: The role of employee proactivity. vol. 54. Academy of Management Journal; 2011.

[16] Griffin MA, Neal A, Parker SK. A New Model of Wrok Role Performance: Positive Behavior in Uncertain and Interdependent Contexts. Academy of Management Journal 2007;50:327–47.

[17] Mazzocco K, Petitti DB, Fong KT, Bonacum D, Brookey J, Graham S, et al. Surgical team behaviors and patient outcomes 2009;197:678–85.

[18] Mishra A, Catchpole K, McCulloch P. The Oxford NOTECHS System: reliability and validity of a tool for measuring teamwork behaviour in the operating theatre. Qual Saf Health Care 2009;18:104–8. doi:10.1136/qshc.2007.024760.

[19] Parker SH, Flin R, McKinley A, Yule S. The Surgeons“ Leadership Inventory (SLI): a taxonomy and rating system for surgeons” intraoperative leadership skills. The American Journal of Surgery 2013;205:745–51. doi:10.1016/j.amjsurg.2012.02.020.

[20] Yule S, Flin R, Paterson-Brown S, Maran N, Rowley D. Development of a rating system for surgeons' non-technical skills. Med Educ 2006;40:1098–104. doi:10.1111/j.1365-2929.2006.02610.x.

[21] Charmaz K. Constructing grounded theory: A practical guide through qualitative research. London: Sage Publications Ltd; 2006.

[22] Singer SJ, Falwell A, Gaba DM, Meterko M, Rosen A, Hartmann CW, et al. Identifying Organizational Cultures that Promote Patient Safety. Health Care Manage Rev 2009;34:300–11.

[23] Attride-Stirling J. Thematic networks: an analytic tool for qualitative research. Qualitative Research 2001;1:385–405.

**TABLES**

| **Supplemental Material-A1: Sample Characteristics for Surveys, Interviews, and Cases Observed** | | | | | |
| --- | --- | --- | --- | --- | --- |
| **Sample Characteristics for Survey** |  |  |  |  |  |
|  | **Team Members** | |  | **Surgeons** | |
|  | **Number** | **%** |  | **Number** | **%** |
| Gender |  |  |  |  |  |
| Male | 31 | 46% |  | 6 | 86% |
| Female | 36 | 53% |  | 1 | 14% |
| Missing | 1 | 1% |  | 0 | 0% |
| Age | | | | | |
| 18 - 29 years | 9 | 13% |  | 0 | 0% |
| 30 - 39 years | 21 | 31% |  | 1 | 14% |
| 40 - 49 years | 10 | 15% |  | 3 | 43% |
| 50 - 59 years | 18 | 26% |  | 2 | 29% |
| 60 - 69 years | 6 | 9% |  | 1 | 14% |
| Missing | 4 | 6% |  | 0 | 0% |
| Hours of work per week | | | | | |
| Less than 20 | 2 | 3% |  | 0 | 0% |
| 20 - 39 | 15 | 22% |  | 0 | 0% |
| 40 - 59 | 38 | 56% |  | 0 | 0% |
| 60 - 79 | 4 | 6% |  | 3 | 43% |
| 80 - 99 | 9 | 13% |  | 1 | 14% |
| 100+ | 0 | 0% |  | 3 | 43% |
| Length of employment at the hospital | | | | | |
| Less than 1 year | 6 | 9% |  | 1 | 14% |
| 1 - 5 years | 28 | 41% |  | 0 | 0% |
| 6 - 10 years | 6 | 9% |  | 2 | 29% |
| 11 - 15 years | 5 | 7% |  | 0 | 0% |
| 16 - 20 years | 7 | 10% |  | 1 | 14% |
| 21 years or more | 15 | 22% |  | 3 | 43% |
| Missing | 1 | 1% |  | 0 | 0% |
| Length of employment in current work area/unit | | | | | |
| Less than 1 year | 9 | 13% |  | 2 | 29% |
| 1 - 5 years | 28 | 41% |  | 1 | 14% |
| 6 - 10 years | 6 | 9% |  | 1 | 14% |
| 11 - 15 years | 7 | 10% |  | 0 | 0% |
| 16 - 20 years | 4 | 6% |  | 2 | 29% |
| 21 years or more | 12 | 18% |  | 1 | 14% |
| Missing | 2 | 3% |  | 0 | 0% |
| Staff position | | | | | |
| Anesthesiologists | 13 | 19% |  |  |  |
| Anesthesia nurses | 6 | 9% |  |  |  |
| Circulating nurses | 9 | 13% |  |  |  |
| Scrub nurses | 13 | 19% |  |  |  |
| Surgical technicians | 4 | 6% |  |  |  |
| Perfusionists | 10 | 15% |  |  |  |
| Physician assistants | 4 | 6% |  |  |  |
| Surgical trainees | 7 | 10% |  |  |  |
| Missing | 2 | 3% |  |  |  |
| Total N | 68 | 100% |  | 7 | 100% |
| **Sample Characteristics for Interviews** | | | | | |
| Staff position | | | | | |
| Anesthesiologists | 3 | 13% |  |  |  |
| Nurses | 8 | 35% |  |  |  |
| Perfusionists | 4 | 17% |  |  |  |
| Physician assistants | 4 | 17% |  |  |  |
| Surgical trainees | 4 | 17% |  |  |  |
| Total N | 23 | 100% |  | 11 | 100% |
| **Sample Characteristics for Cases Observations** | | | | | |
|  | Average | |  | Range | |
| Length of procedure, hours | 5 | |  | 2-9 | |
|  | | | | | |
| Procedure type | Number | |  | % | |
| Aortic valve replacement | 10 | |  | 45% | |
| Coronary artery bypass graft | 6 | |  | 27% | |
| Mitral valve replacement | 4 | |  | 18% | |
| Carotid artery replacement | 1 | |  | 5% | |
| Heart transplant | 1 | |  | 5% | |
| Total N | 22 | |  | 100% | |

| **Supplemental Material-A2. Survey Questions 1-16 Overall and by Role (Surgeon, Non-Surgeon) - Pre and Post Surveys Combined** | | | | | | | | | | | | | |
| --- | --- | --- | --- | --- | --- | --- | --- | --- | --- | --- | --- | --- | --- |
|  |  |  |  |  |  |  |  |  |  |  |  |  |  |
| Measure | PRE + POST SURVEY Combined | | | | | | | | | | | | |
|  | Overall | | |  | Surgeons | | |  | Non-Surgeons | | |  | P Value  Surgeons vs Non-Surgeons |
|  | N | Mean | SD |  | N | Mean | SD |  | N | Mean | SD |  |  |
| **Self Efficacy** | 75 | 6.51 | 0.67 |  | 7 | 6.90 | 0.19 |  | 68 | 6.47 | 0.69 |  | 0.000 |
| **Social Worth** | 75 | 5.48 | 1.09 |  | 7 | 6.29 | 0.62 |  | 68 | 5.39 | 1.09 |  | 0.037 |
| Job Satisfaction | 75 | 5.03 | 1.48 |  | 7 | 5.14 | 2.17 |  | 68 | 5.01 | 1.41 |  | 0.829 |
| Burnout/Emotional Exhaustion | 75 | 4.20 | 1.60 |  | 7 | 4.26 | 1.64 |  | 68 | 4.19 | 1.61 |  | 0.915 |
| **Generalized Sense of Power Scale** | 75 | 4.74 | 1.19 |  | 7 | 5.95 | 1.06 |  | 68 | 4.62 | 1.13 |  | 0.004 |
| **Perceived Power** | 75 | 3.59 | 1.26 |  | 7 | 5.14 | 1.21 |  | 68 | 3.43 | 1.17 |  | 0.000 |
| **Perceived Status** | 75 | 4.89 | 1.26 |  | 7 | 6.05 | 1.05 |  | 68 | 4.77 | 1.22 |  | 0.009 |
| Team Identification | 74 | 5.74 | 0.98 |  | 7 | 5.90 | 1.13 |  | 67 | 5.72 | 0.97 |  | 0.640 |
| **Psychological Safety** | 74 | 4.16 | 1.30 |  | 7 | 5.19 | 1.72 |  | 67 | 4.05 | 1.21 |  | 0.026 |
| **Open Communication** | 75 | 4.49 | 1.51 |  | 7 | 6.05 | 0.89 |  | 68 | 4.33 | 1.48 |  | 0.004 |
| Coworker relationship quality | 75 | 5.00 | 1.34 |  | 7 | 5.19 | 1.31 |  | 68 | 4.98 | 1.36 |  | 0.690 |
| Individual learning | 74 | 5.80 | 0.77 |  | 7 | 5.62 | 0.67 |  | 67 | 5.82 | 0.78 |  | 0.520 |
| Team learning | 74 | 4.70 | 1.40 |  | 7 | 4.93 | 1.22 |  | 67 | 4.68 | 1.42 |  | 0.654 |
| Team confidence | 74 | 5.88 | 1.01 |  | 7 | 5.93 | 0.77 |  | 67 | 5.88 | 1.04 |  | 0.896 |
| Overall rating for team | 74 | 5.19 | 1.32 |  | 7 | 5.43 | 0.84 |  | 67 | 5.16 | 1.36 |  | 0.618 |
| Notes: | | | | | | | | | | | | | |
| All mean scores based on 1-7 scale | | | | | | | | | | | | | |
|  | | | | | | | | | | | | | |

**Supplemental Material-A3. Behavior types by leadership function with a description and examples.**

| ***Leadership function/*** Behavior | **Description** | **Valence** | **Example 1** | **Example 2** |
| --- | --- | --- | --- | --- |
| ***Elucidator*** | | | | |
| Teaching | Teaching another team member how to do something. Calling attention to something worthy of note to create an opportunity for a team member to learn/practice. Includes technical teaching, pertaining to science, surgery, medicine, body; and non-technical teaching, pertaining to communication, leadership, teamwork, interactions. | + | Surgeon to Fellow: “It is the nature of this trade. Everything needs to be fine and precise. You need to visualize what you’re doing before you do it. You don’t just put a stitch in then take it out.” | Surgeon to Fellow: "when you are about to do this maneuver, you want to ask your scrub for X in Y way, that way she will know to give you Z." |
| Constructive criticism | Providing negative feedback in a constructive manner. | + | Surgeon to scrub: "This operating field is not clear. These tools are getting in my way. Scrub, even if it takes an extra second, could you please make sure the field is clear as soon as the valve is in next time? Thanks. " | Surgeon to Fellow after Fellow motioned to close the chest: "We absolutely never close the chest until the count is complete. Please, make sure to wait for the completed count before beginning to close." |
| Private criticism | Critiquing or scolding on the side (not for the entire room to hear). | - | Surgeon to Fellow in the back of the room, "You have got to start implementing the lessons I've taught you. Do you understand?" | Surgeon to Perfusionist: "We talked about that this morning! Meet me outside the OR after the case and I will make it clear again." |
| Negative criticism | Providing negative feedback. | - | Surgeon to Fellow: "What are you doing? You’re trying to defy biology by doing that. You must never do that again! How are you ever going to improve?” | Surgeon to Fellow: "Take the stuff we tell you about and do it NOW. Not tomorrow. Not a year from now. NOW! STOP. Practice this at home. That is not something you should do on a person." |
| Explanation | Speaking aloud to inform others how he/she perceives what’s going on; thinking or interpreting aloud; providing reasoning for decisions or their outcomes. | + | Surgeon to whole OR: "We are going to do this sternotomy just like we do redos even though this is not a redo; this line will go here, this line here, and this line here." | Surgeon to Fellow, "We're going to go forward with the replacement instead of the repair. This valve [tissue] is weaker than I expected. I see signs of failure already. It won't hold with a repair." |
| Relevance giving | Adding "the because" to requests or interpreting data aloud for others to hear. | + | Perfusionist shared information about the pressure and flow rate and surgeon responded, "wow, high, huh? That's likely because of X. Thanks." | Surgeon to Perfusionist: "Raise the pressure…because we are testing the anastomosis.” |
| ***Tone setter*** | | | | |
| Conversation unrelated to the case | Friendly discussion about personal/non-work related issues, occurring during non-critical periods in the case. | o | Surgeon to Scrub: "Have you ever travelled to India? I went last year and it was amazing." | Surgeon to Fellow: "Did you see that movie? Did you like it?" |
| Constructive humor | Making an inclusive joke. Could include self-deprecating humor. | + | Surgeon to whole OR: "Pardon my strength!" | Surgeon to whole OR: "Man, look at this audience in my OR. It's like they're coming to watch Katy Perry or something!" |
| Compliment | Saying something nice about someone else’s work. | + | Surgeon to Perfusionist after difficulties with drainage earlier in the case: “your drainage is perfect now! Great job.” | Medical Student responds to Surgeon's quiz question and surgeon says: "Nice. You have your physiology down pat." |
| Reassurance | Saying comforting or forgiving words in response to a mistake made by a team member. | + | Surgeon to Medical Student who has been holding back the skin for a while: "Don't worry, your torture will be over soon, I promise." | Scrub broke something. Surgeon to Scrub, "Don't worry. Mistakes happen. It's fine." |
| Encouragement | Sharing reassuring words with the room. | + | Surgeon to whole OR: “Well, glad we’re underway! That could have been bad.” | Surgeon to whole OR: "Almost there. Almost there!" |
| Frustration | Expressing annoyance or anger. | - | Surgeon to Fellow: "You've got to cut the sternum straight. I've told you this a thousand times!" | Surgeon to Fellow [Fellow sewing too slowly]: "Oh, come on!" |
| Destructive humor | Sarcasm, demeaning statements, joke at the expense of patient or provider. | - | Surgeon to Scrub Nurse: "Do you think I should just go into the heart with my bare hands [without gloves]. Do you think that would be bad judgment?” | Surgeon to Circulating Nurse: "You get a 94% for the day. Not 100% because you handed me that twisted suture." |
| ***Engagement Facilitator*** | | | | |
| Collaboration | Working with another team member to make a decision or interpret data, information, or observations. | + | Surgeon to Physician Assistant: "Will you have enough vein, [Physician Assistant]?" Physician Assistant: "Yes, I think so." Surgeon: "Okay great. What's the condition of the vein? Do you think we should use it for the bypass?" | Surgeon to Anesthesiologist: "How does the new valve look?" Anesthesiologist to surgeon: "We see some leakage from the left side." Surgeon to Anesthesiologist: "If I repair that side, how do you think the right side will look?" Anesthesiologist: "Good from our end." |
| Consultation | Asking another team member if it’s okay to proceed or for a status update from their perspective. | + | Surgeon to Anesthesiologist: "How does the valve look? Any leakage? Does it look well sealed? If so, I'll close the chest." | Surgeon to Perfusionist: "Are you fully warmed? If so, we're ok to come off bypass." |
| Helping/supporting | Contributing to the group's work and/or lending assistance to another team member. | + | Circulating Nurse tries to get attention of the room for the checklist. Everyone keeps talking and doesn't pay attention. Surgeon says loudly, "Quiet everyone, we're going to do the checklist now!" | Surgeon to Physician Assistant: "Here, I'll work on doing X part of the case so that I'm out of your way as you take the vein out of the leg." |
| Apology | Expressing regret for a mistake or a misunderstanding. | + | New Fellow was giving the Surgeon an update and the Surgeon needed to interrupt her to give a time-sensitive command to the Perfusionist so he said, “sorry could I interrupt you for a second?” He then gave the command to the Perfusionist. He then turned back to the Fellow and said, "Okay, go on, thanks." | After some confusion surrounding pressure levels, Surgeon to Perfusionist: "Sorry about that guys." |
| Thanks | Expressing appreciation to another for his/her help or job well done. | + | Surgeon to Circulating Nurse after she adjusted the surgeons headlight: "Thanks very much for your help." | As Circulating Nurse walks out of the room (because Circulating Nurse is handing off), surgeon says: "Thanks for your help today." |
| Inquiry | Question to identify potential problem or to indicate concern. | + | Surgeon to Anesthesiologist: "Your line looks damp, guys. Is there something wrong with it?" | Surgeon to Anesthesiologist: "What's the large spike on the monitor? Do you see it? What could have caused that sudden increase in pressure?" |
| ***Delegator*** | | | | |
| Request | Giving instruction to a team member to do something. | 0 | Surgeon to Scrub Nurse: "Open two surgiflows, please, then hand me the forceps." | Surgeon to Circulating Nurse: "Page Dr. X to see if he needs a sample of this artery." |
| Help Seeking | Asking for assistance from others. | + | Surgeon to Anesthesiologist: "Could you take a look at the new valve and let me know if there are any leaks?" | Surgeon to Fellow, "Could you come on this side of the table and assist with the LIMA?" |
| ***Safe space maker*** | | | | |
| Non-surgeon initiated concern | Team member raises a safety or quality concern. | + | Perfusionist to Surgeon: "I just want to make sure that you really do want the auto-inflate retrograde catheter instead of the manually inflated one." | Scrub Nurse to Surgeon: "Dr. X, we just finished our count but we are short one needle…!" |
| Non-surgeon initiated questioning | Team member asks a question without prompting from the surgeon. | + | Circulating Nurse to Surgeon: "Just checking, do you still need the sucker turned on or can I clear it out of the way to make space for the freezing machine?" | Scrub Nurse to Surgeon: "What are you thinking about when you are deciding whether to do a repair vs. replacement?" |
| Non-surgeon information sharing | Team member shares information without prompting from the surgeon. | + | Anesthesiologist to Surgeon: "[Surgeon's name], we see moderate MR on the ECHO coming from the left side." | Circulating Nurse to Surgeon: "Dr. X, the Pathologist is on the phone and he says the sample results came back negative." |
| ***Conductor*** | | | | |
| Focus returning | Announcing to the room that it is time to focus on case-related matters. | + | Surgeon to the whole OR: “Okay guys, we’re going to do another checklist now. Listen up!” | Surgeon to big group of Anesthesiologists that just walked into the room, chatting loudly: "Hey, could you guys keep it down a bit back there? We're going through a tricky part." |
| Concern anticipation | Identifying for team members a likely outcome before it occurs in order to create expectations and alleviate potential worry. | + | Surgeon to the whole OR: "I don't think this will happen, but there's a chance we'll have to go on bypass really quickly if the hematoma bursts so let's all be ready just in case." | Surgeon to Circulating Nurse: “I might need microsutures, but I am not sure yet. But please just have them in the room in case.” |
| Step mapping | Describing in advance for the room to hear the next few steps in the procedure. | + | Surgeon to scrub nurse: "The first stitch will be forehand with a felted suture, the second stitch will be backhand with a non-felted suture, the third stitch will be forehand with the felted suture." | Surgeon to the whole OR: "Okay, we're going to put in the retrograde, then do the mitral valve, then the AV valve, then the rest. Okay? Sound good?" |
| Loop closing for confirmation | Structured back and forth communication between the surgeon and another team member, in which the surgeon requests a task and the team member repeats to confirm the task has been completed. | + | Surgeon to Perfusionist: "Pressure up." Perfusionist to surgeon: "Pressure is up." Surgeon to perfusionist: "Increase the flow." Perfusionist to surgeon: "Flow is increased." | Surgeon to Anesthesiologist: "Administer protamine." Anesthesiologist: "Okay, giving protamine now." |
| Need for clarification | Request by surgeon that prompts a team member to follow up with a repetition of that request, phrased as a question indicating that original request was not understood. | - | Surgeon to Perfusionist: "Full flow for uno momento por favor." Perfusionist: "Huh? How long?" Surgeon to Perfusionist: "For one minute, sorry." | Surgeon to Perfusionist: "Pressure up." Perfusionist to Surgeon: "Was that directed at me?" |
| ***Being human*** | | | | |
| Showing fatigue | Showing evidence of stress/strain on the body. | o | Surgeon to Circulating Nurse: “Can you let upstairs know that this case is trying to kill me. It will never be done. I won’t make the 1pm appointment.” | Surgeon takes a pause, stretches out his neck and rolls his shoulders with eyes closed. Then returns to case. |
| Musing | Observation of more generalizable nature; may be sarcastic. | o | Surgeon to whole operating room: “Hope springs eternal. You gotta believe!” | Surgeon to Fellow: "A good dissector is like Christopher Columbus or Vasco de Gama. They always knew where they were!" |
| Self-questioning | Sharing uncertainty about one's own performance. | + | Surgeon to himself/OR: "We want to increase the flow into the left side of the heart...That's the blue line, right?" Perfusionist confirmed, “yup, the blue one.” | Surgeon to Scrub Nurse: "Four more after this, right?" |
| Jargon | Speaking in idiosyncratic terms. | - | Surgeon to Fellow: "We've hit the landmine!" | Surgeon to Perfusionist: "We're going to do the deep dive!" |

**Supplemental Material-A4. Comparison of leadership function and behavior valence for the two highest and two lowest ranked surgeons**

|  | **Two highest ranked surgeons^** | | | **Two lowest ranked**  **surgeons^** | | |
| --- | --- | --- | --- | --- | --- | --- |
| Domain | **N** | **% of all behaviors** | **% of given function** | **N** | **% of all behaviors** | **% of given function** |
| **Leadership function***** |  |  |  |  |  |  |
| *Elucidator* | 158 | 24.9% |  | 159 | 35.7% |  |
| Positive: constructive criticism, teaching, explanation, relevance giving | 143 |  | 90.5% | 84 |  | 52.8% |
| Negative: negative criticism, private criticism | 15 |  | 9.5% | 75 |  | 47.2% |
| *Tone setter* | 140 | 22.0% |  | 137 | 30.7% |  |
| Positive: constructive humor, compliment, encouragement, reassurance | 85 |  | 60.7% | 26 |  | 19.0% |
| Neutral: conversation unrelated to the case | 34 |  | 24.3% | 53 |  | 38.7% |
| Negative: destructive humor, frustration | 21 |  | 15.0% | 58 |  | 42.3% |
| *Engagement facilitator:* collaboration, consultation, helping/supporting, apology, thanks, inquiry | 121 | 19.1% |  | 32 | 7.2% |  |
| *Safe space maker:* non-surgeon initiated concern, non-surgeon initiated questioning, non-surgeon information sharing | 97 | 15.3% |  | 47 | 10.5% |  |
| *Delegator* | 69 | 10.9% |  | 43 | 9.6% |  |
| Positive: help seeking | 3 |  | 4.3% | 0 |  | 0.0% |
| Neutral: request | 66 |  | 95.7% | 43 |  | 100.0% |
| *Conductor* | 38 | 6.0% |  | 23 | 5.2% |  |
| Positive: concern anticipation, focus returning, loop closing for confirmation, step mapping | 36 |  | 94.7% | 22 |  | 95.7% |
| Negative: clarification | 2 |  | 5.3% | 1 |  | 4.3% |
| *Being human* | 12 | 1.9% |  | 5 | 1.1% |  |
| Positive: self-questioning | 1 |  | 8.3% | 1 |  | 20.0% |
| Neutral: musing, showing fatigue | 8 |  | 66.7% | 4 |  | 80.0% |
| Negative: jargon | 3 |  | 25.0% | 0 |  | 0.0% |
| **Behavior valence***** |  |  |  |  |  |  |
| *Positive* | 486 | 76.5% |  | 212 | 47.5% |  |
| *Neutral* | 108 | 17.0% |  | 100 | 22.4% |  |
| *Negative* | 41 | 6.5% |  | 134 | 30.0% |  |
| Notes: ^ Performance based on non-surgeon perception of surgeon’s effectiveness as a team leader; counts and percentages in the table represent pooled estimates of all behaviors observed among the two surgeons in each group (high and low performance) and thus reflect differences in total behaviors observed per surgeon. | | | | | | |
| ***p<0.0001 | | | | | | |

**Supplemental Material-A5. Correlation between leadership function (observations) and non-surgeon perception of surgeon as team leader (survey)**

| **Leadership function** | **Valence of behavior type** | **Correlation** | **P-value** |
| --- | --- | --- | --- |
| Engagement facilitator | Positive | **0.80** | **0.03** |
| Conductor | Positive | 0.03 | 0.96 |
|  | Negative | 0.27 | 0.56 |
| Tone setter | Positive | 0.62 | 0.14 |
|  | Negative | **-0.68** | **0.09** |
| Elucidator | Positive | 0.26 | 0.58 |
|  | Negative | **-0.81** | **0.03** |
| Delegator | Positive | 0.37 | 0.41 |
| Being human | Positive | 0.04 | 0.93 |
|  | Negative | 0.22 | 0.64 |
| Safe space maker | Positive | 0.54 | 0.22 |
| All Interactions | Positive | **0.85** | **0.02** |
|  | Negative | **-0.750** | **0.05** |

**FIGURES**

**Supplemental Material-Figure 1. Conceptual framework of surgeon leadership**

Perceptions of effective team leadership

Leadership functions

Behaviors

Operating environment

Note: See Supplemental Material-A3 for a list of the forms of behaviors grouped into leadership functions, with description and examples for each form of behavior.
