# Supplementary material for: Effective Leadership of Surgical Teams: A Mixed Methods Study of Surgeon Behaviors and Functions
Source: Ann Thorac Surg. 2017 Aug;104(2):530–7. doi: 10.1016/j.athoracsur.2017.01.021 (PMC5527126; doi:10.1016/j.athoracsur.2017.01.021)
Supplement: Supplemental Material-B [file mmc3.docx]

**ATTACHMENT 1: SURGICAL TEAM MEMBER PRE AND POST SURVEY**

**[INSTRUCTIONS]** In this survey, you will be asked to answer some questions about yourself and the surgical teams with which you work. For the questions regarding your “team”, please think about all the teams you work with in cardiac surgery, and answer the questions with reference to your average experience among them.

**[INSTRUCTIONS]** The first few questions ask you about yourself and your job.

**[Big 5 personality traits]**

Here are a number of personality traits that may or may not apply to you. Please write a number next to each statement to indicate the extent to which you agree or disagree with that statement. You should rate the extent to which the pair of traits applies to you, even if one characteristic applies more strongly than the other.

| 1 | 2 | 3 | 4 | 5 | 6 | 7 |
| --- | --- | --- | --- | --- | --- | --- |
| Strongly Disagree | Moderately Disagree | Disagree A Little | Neither Agree nor Disagree | Agree A Little | Moderately Agree | Strongly Agree |

I see myself as:

1. _____ Extraverted, enthusiastic.

2. _____ Critical, quarrelsome.

3. _____ Dependable, self-disciplined.

4. _____ Anxious, easily upset.

5. _____ Open to new experiences, complex.

6. _____ Reserved, quiet.

7. _____ Sympathetic, warm.

8. _____ Disorganized, careless.

9. _____ Calm, emotionally stable.

10. _____ Conventional, uncreative.

TIPI scale scoring (‘‘R’’ denotes reverse-scored items): Extraversion: 1, 6R; Agreeableness: 2R, 7;

Conscientiousness; 3, 8R; Emotional Stability: 4R, 9; Openness to Experiences: 5, 10R.

**[Self-efficacy]**

Indicate the extent to which you agree/disagree with each of the following statements (1 = completely disagree, 7 = completely agree):

1. I feel capable.

2. I feel competent.

3. I usually make good judgments.

**[Social worth]**

Indicate the extent to which you agree/disagree with each of the following statements (1 = completely disagree, 7 = completely agree):

1. I feel valued as a person by the other team members.

2. I feel appreciated as an individual by the other team members.

3. I think I make a positive difference in people’s life because of what I do.

**[Job Satisfaction]**

Indicate the extent to which you agree/disagree with each of the following statements (1 = completely disagree, 7 = completely agree):

1. If a good friend told me that he/she was interested in working in a job like mine, I would strongly recommend it.

2. All in all, I am very satisfied with my current job.

3. In general, my job measures up to the sort of job I wanted when I took it.

**[Burnout/Emotional Exhaustion]**

Indicate the extent to which you agree/disagree with each of the following statements (1 = completely disagree, 7 = completely agree):

1. I feel emotionally drained from my work.

2. I feel used up at the end of the workday.

3. I feel burned out from my work.

**[INSTRUCTIONS]** The next few questions ask you about your team.

**[Generalized Sense of Power Scale]**

Indicate the extent to which you agree/disagree with each of the following statements (1 = completely disagree, 7 = completely agree):

In my relationships with my team members in the OR where I work…

1. I can get them to listen to what I say.
2. Even if I voice them, my views have little sway.
3. My ideas and opinions are often ignored.

**[Perceived Power]**

Please think about the last experience you had interacting with team members in the operating room. What amount of power did you personally feel in that experience? (scored 1=very little to 7=a great deal).

**[Perceived Status]**

Indicate the extent to which you agree/disagree with each of the following statements (1 = completely disagree, 7 = completely agree):

In the ORs where I work…

1. I am confident that my team members respect me.
2. I have high status in my team members’ eyes.
3. I have influence over my team members’ behavior.

**[Team Identification]**

Indicate the extent to which you agree/disagree with each of the following statements (1 = completely disagree, 7 = completely agree):

1. When I walk into the operating room, I see the group of people I am working with as my team.

2. When I walk into the operating room, I think of the surgical staff as my team.

**[Psychological safety]**

Indicate the extent to which you agree/disagree with each of the following statements (1 = completely disagree, 7 = completely agree):

1. When I make a mistake in this team, it is often held against me.

2. I am able to bring up problems and tough issues.

3. It is safe for me to take a risk in this team.

**[Open communication]**

Indicate the extent to which you agree/disagree with each of the following statements (1 = completely disagree, 7 = completely agree):

1. All members had a chance to express opinions.

2. Team members listened to each other’s input.

3. Members were free to make positive and negative comments.

**[Coworker relationship quality]**

Indicate the extent to which you agree/disagree with each of the following statements (1 = completely disagree, 7 = completely agree):

1. I have close relationships with my team members.

2. I have trusting relationships with my team members.

3. I have open relationships with my team members.

**[Individual learning]**

Indicate the extent to which you agree/disagree with each of the following statements (1 = completely disagree, 7 = completely agree):

1. I adapt well to changes in core tasks.

2. I respond constructively to changes in the way my team works.

3. I easily learn new ways to execute my job more effectively.

**[Team learning]**

Indicate the extent to which you agree/disagree with each of the following statements (1 = completely disagree, 7 = completely agree):

1. In my experience, my team adapts well to changes in core tasks.

3. In my experience, my team responds constructively to changes in the way it works.

4. In my experience, my team easily learns new ways to execute the job more effectively.

This questions seemed perhaps the least necessary to edit. Could also say, “in my opinion” or “I believe” or “I have seen” or no change.

**[Team confidence]**

Indicate the extent to which you agree/disagree with each of the following statements (1 = completely disagree, 7 = completely agree):

1. I am confident that my team works effectively.

2. I feel confident in my team’s abilities.

3. I feel confident that my team provides safe care.

**[Additional surgeon-specific questions]**

Please answer each of the following questions for each of the types of team members with whom you work:

When I code this in qualtrics, will I have a specific page for each team member type? Will they preselect the kinds of people they work with and will I use that to prepopulate the survey?

**Innovation/Taking Charge**

Indicate the extent to which you agree/disagree with each of the following statements (1 = completely disagree, 7 = completely agree):

1. In my experience, this person often tries to adopt improved ways for doing his or her job.

2. In my experience, this person often makes constructive suggestions for improving surgical care.

3. In my experience, this person often tries to implement solutions for problems we encounter in the operation room.

**Quality/In-Role Performance**

Indicate the extent to which you agree/disagree with each of the following statements (1 = completely disagree, 7 = completely agree):

1. In my opinion, this person has mastered the responsibilities in his/her role.

2. In my opinion, this person performs all the tasks that are expected as part of his/her role.

3. This person meets my performance expectations.

**Overall rating**

1. How would you evaluate the general performance of this person?

Poor 1 2 3 4 5 Excellent

**Learning assessment**

Indicate the extent to which you agree/disagree with each of the following statements (1 = completely disagree, 7 = completely agree):

1. In my experience, this person adapts well to changes in core tasks.

2. In my experience, this person responds constructively to changes in the way his/her team works.

3. In my experience, this person easily learns new ways to execute his/her job more effectively.

**Overall rating for team**

1. How would you evaluate the teamwork of your surgical team?

Poor 1 2 3 4 5 Excellent

These are in the present tense while the team-related questions below are in the past tense. Does this matter?

**[Additional team members-specific questions]**

Please answer each of the following questions asking you to evaluate each of the seven surgeons:

**[Assessment surgeon’s approach (e.g., dominance and openness)]**

Indicate the extent to which you agree/disagree with each of the following statements (1 = completely disagree, 7 = completely agree):

1. In my experience, the surgeon was open to new ideas.

2. In my experience, the surgeon was receptive to suggestions.

3. In my experience, the surgeon was interested in our perspective.

4. In my experience, the surgeon seemed to just want everyone to obey him/her.

5. In my experience, the surgeon made me feel pressure.

6. In my experience, the surgeon scolded other team members.

Do we want to say “in my experience here?” Or something like, “the last time I worked with this surgeon…” The above is in past tense whereas the surgeon-specific questions are in the present tense.

**Overall rating**

1. How would you evaluate the general performance of this surgeon as a team leader?

Poor 1 2 3 4 5 Excellent

2. How would you evaluate the teamwork of your surgical team?

Poor 1 2 3 4 5 Excellent

**Demographic information**

This information will help in the analysis of the survey results. Mark ONE answer by filling in the circle.

1. How long have you worked in this hospital?

| 🌕 a. Less than 1 year | 🌕 d. 11 to 15 years |
| --- | --- |
| 🌕 b. 1 to 5 years | 🌕 e. 16 to 20 years |
| 🌕 c. 6 to 10 years | 🌕 f. 21 years or more |

2. How long have you worked in your current hospital work area/unit?

| 🌕 a. Less than 1 year | 🌕 d. 11 to 15 years |
| --- | --- |
| 🌕 b. 1 to 5 years | 🌕 e. 16 to 20 years |
| 🌕 c. 6 to 10 years | 🌕 f. 21 years or more |

3. Typically, how many hours per week do you work in this hospital?

| 🌕 a. Less than 20 hours per week | 🌕 d. 60 to 79 hours per week |  |
| --- | --- | --- |
| 🌕 b. 20 to 39 hours per week | 🌕 e. 80 to 99 hours per week |  |
| 🌕 c. 40 to 59 hours per week | 🌕 f. 100 hours per week or more |  |

4. What is your staff position in this hospital? Mark ONE answer that best describes your staff position.

a. Surgeon

b. First Assistant

c. Anesthesia provider

d. Surgical nurse

e. Surgical technician

f. Perfusionist

g. Circulating nurse

5. What is your gender?

🌕 a. Male

🌕 b. Female

6. What is your approximate age?

| 🌕 a. 18 to 29 | 🌕 d. 50 to 59 |
| --- | --- |
| 🌕 a. 30 to 39 | 🌕 d. 60 to 69 |
| 🌕 a. 40 to 49 | 🌕 d. 70 or older |

***THANK YOU FOR COMPLETING THIS SURVEY.***
