# Supplementary material for: Effective Leadership of Surgical Teams: A Mixed Methods Study of Surgeon Behaviors and Functions
Source: Ann Thorac Surg. 2017 Aug;104(2):530–7. doi: 10.1016/j.athoracsur.2017.01.021 (PMC5527126; doi:10.1016/j.athoracsur.2017.01.021)
Supplement: Supplemental Material-C [file mmc4.pdf]

Procedure Performed: ______________________________ **Surgeon Code:** ____________________________

Cardiac Surgery Observation Tool	
Date of Procedure	___/___/_____
Time of Incision	___:___ AM/PM
Surgeon Arrival Time	___:___ AM/PM
Surgeon Departure Time	___:___ AM/PM
Surgical End Time	___:___AM/PM
Case delay	_______ minutes
Room Number	________________

Ask Circulating Nurse			
Level of Difficulty	☐ Below Average	☐ Average	☐ Above Average
Outcome of Patient	☐ Worse	☐ As Expected	☐ Better
Deviation from Operative Plan	☐ Yes ☐ No		
Deviation from Regular Behavior	☐ Yes ☐ No		
Video Shown	☐ Yes ☐ No		

Surgical Checklist Performed	☐ Pre-Operative	☐ Before Incision	☐ Before Perfusion	☐ After Perfusion	☐ Before Patient Leaves OR
Surgeon Involved (circle)	Yes/No	Yes/No	Yes/No	Yes/No	Yes/No

1st Asst./Resident M/F	Anesthesia M/F	Scrub M/F	Perfusionist M/F	Circulating Nurse M/F	M/F	No one in particular

1st Asst./Resident	M/F	Anesthesia	M/F	Scrub	M/F	Perfusionist	M/F	Circulating Nurse	M/F		M/F	No one in particular

Code Book and Quantitative Comment Tally

Surgeon Comment Tally	Comment Type	Description	Example
	**Self-Questioning**	Sharing uncertainty about one's own performance.	"I don't know whether I can trust this thing when I am finished."
	**Humor**	Can be positive or negative.	Positive: Making a joke, laughing at a joke<br>Negative: Sarcasm, demeaning statements, joke at the expense of a patient or provider
	**Explanation**	Speaking out loud to inform others how he/she perceives what's going on.	
	**Compliment**	Saying something nice about someone else's work.	"Nice vein."
	**Consultation**	Asking if it's okay to proceed. Asking for status update from another's perspective.	
	**Inquiry**	Question to identify potential problem or indicate concern.	
	**Help Seeking**	Asking for support from others.	"Speak to me."
	**Teaching**	Teaching another staff member how to do something. Calling attention to something worthy of note. Allowing opportunity for resident to learn/practice.	
	**Small Talk**	Friendly discussion about personal/non-work related issues.	"Announcer just said Justin Bieber instead of Justin Verlander."
	**Pontification**	Observation of more generalizable nature; may be sarcastic.	"Failing to prepare is preparing to fail."<br>"We're earning our salary here today."
	**Private Instruction**		Doctor scolded Snr. Perfusionist for not being present at pre-op checklist to supervise student, according to Snr. Perfusionist. He was upstairs caring for another patient but formally it was not appropriate.
	**Fatigue**	Evidence of stress/strain on the body.	
	**Thanks**		
	**Direction/Command**	Giving instruction to do something.	"Tilt the table back to the center."
	**Criticism**	Can be either constructive or without constructive suggestion.	Without: "Don't pull it like that."<br>With: "Don't pull it like that. Move the vein in this direction instead."

Debrief

1.) During the period of surgeon arrival to the point when the main elements of the procedure are complete, to what extent do you think the team interactions showed:

5	4	3	2	1
Good rapport with everyone		Neutral or mixed, person-specific rapport		Bad rapport with everyone

5	4	3	2	1
Very collaborative environment with everyone		Neutral or mixed, person-specific collaboration		Speak when spoken to or not at all (applied to everyone)

5	4	3	2	1
Very relaxed		Neutral		Very tense

2.) How did the room feel? What was the dynamic like?

3.) What were the strengths around team communication, interactions, and dynamics?

4.) Any concern regarding team interaction and communication?

5.) Anything else?
