# Supplementary material for: Effective Leadership of Surgical Teams: A Mixed Methods Study of Surgeon Behaviors and Functions
Source: Ann Thorac Surg. 2017 Aug;104(2):530–7. doi: 10.1016/j.athoracsur.2017.01.021 (PMC5527126; doi:10.1016/j.athoracsur.2017.01.021)
Supplement: Supplemental Material-D [file mmc5.docx]

**Pretest Interview Protocol**

Effective Leadership and Team Dynamics in Surgical Teams

Protocol Number: 2013P001500

Thank you for agreeing to talk with me today. You have been selected to speak to us because you are a part of the [insert discipline here] team. Our research project is focused on identifying areas for improvement in the way team members interact and learn from one another during surgery. More specifically, we would like to better understand current levels of teamwork and how teamwork relates to other individual and interpersonal factors. For the questions regarding your “team”, please think about all the operating room teams you work with in cardiac surgery, and answer the questions with reference to your average experience among them. By team, we mean the group of people you work with in the operating room, including surgeons, fellows, residents, physician assistants, anesthesiologists, anesthesia nurses, perfusionists, scrub nurses, circulating nurses, and surgical technicians. Through this interview, we would like to hear from you, hear your thoughts on team dynamics in the OR and what you would like to see done differently or more consistently in the OR.

Your participation in this study is purely voluntary and you may stop interviewing at any point or refuse to respond to any questions that you don’t feel comfortable answering. Your participation in this interview will not be compensated. We are scheduled to talk with you for about fifteen minutes. Does this timing still work with your schedule?

I also would like to ask for your permission to tape record our conversation. Tape recording is optional. This is only to ensure that I will capture everything that we discuss. I will not share this tape with anyone outside of our research team, and your name will not be identified in any publications or presentations. Audio recordings will be stored only until transcribed – at maximum 18 months, which is the duration of the study – and then will be destroyed.

Do you feel comfortable being audio recorded? (Circle one) Yes No

**Interviewee Name and Title: __________________________________**

**Interviewee Signature: _______________________________________**

**Interviewer (s): _____________________________________________**

Questions

1. How would you characterize team dynamics in the cardiac surgery operating rooms (OR’s) at its best?  About what percent of the time do you experience this dynamic?
2. How would you characterize team dynamics in the cardiac surgery OR’s at its worst? About what percent of the time do you experience this dynamic?
3. What factors tend to determine whether OR team dynamics are better or worse?
4. If you could change one thing about the way people interact in the cardiac surgery ORs, what would it be?
5. Do you have any recommendations for how to accomplish this change?
6. Repeat 4 and 5 (I.e., If you could change one more thing…?
7. Is there anything else you would like to discuss in terms of team dynamics in the cardiac surgery OR’s?

Thank you so much for taking the time out to speak to us today. Please don’t hesitate to contact me if you have any questions. Is it okay if I contact you if we need any additional information?

**Posttest Interview Protocol**

Effective Leadership and Team Dynamics in Surgical Teams

Protocol Number: 2013P001500

Thank you for agreeing to talk with me today. We’re speaking with you as a member of the [insert discipline here] team for the follow up phase of our research project about the way team members interact and learn from one another during surgery. We’re interested in understanding your impressions of current levels of teamwork and how teamwork may have changed in the last year.

Your participation in this study is purely voluntary and you may stop interviewing at any point or refuse to respond to any questions that you don’t feel comfortable answering. Your participation in this interview will not be compensated. We are scheduled to talk with you for about thirty to forty-five minutes. Does this timing still work with your schedule?

I also would like to ask for your permission to tape record our conversation. Tape recording is optional. This is only to ensure that I will capture everything that we discuss. I will not share this tape with anyone outside of our research team, and your name will not be identified in any publications or presentations. Audio recordings will be stored only until transcribed – at maximum 18 months, which is the duration of the study – and then will be destroyed.

Do you feel comfortable being audio recorded? (Circle one) Yes No

**Interviewee Name and Title: _________________________________________**

**Interviewee Signature: _______________________________________________**

**Interviewer (s): ______________________________________________________**

Questions:

*To start off, a few general questions.*

1. Have you had the opportunity to learn about our study findings thus far?
2. Who would you normally consider to be part of your team?
3. To what extent do you think people in other surgical roles understand what your job involves and what you need to do it well? And vice versa…
4. What would team dynamics be like in your ideal OR? Do you think others have the same ideal picture of team dynamics?

*Second interview – asking about any changes you’ve seen over period we’ve been doing study*

1. Have you noticed any changes in the extent to which you find team dynamics in the cardiac surgery ORs here to be positive?
2. Have you observed any behavior changes among the surgeons in the past year?
   1. Which change(s) if any have you found to be most effective?
   2. How do you suggest the change(s) be solidified?
3. For you personally, what change would make you more excited to get up and operate with these surgeons everyday?

*Another thing we thought would be useful, feedback abt our findings, interpretation as its emerging.*

1. We have observed that sharing case-relevant information between the surgeon and his teammates is a productive form of communication. Is more “information sharing” between the surgeon and his/her teammates part of your vision of when the OR is at its best?
2. We’d like to ask you a few specific questions about things we observed in the OR:
   1. Do you find small talk and chitchat to be comforting or distracting during cases?
   2. What style and tone of teaching (BY SURGEONS) do you find most useful?
   3. Do you find yourself needing to clarify requests often or are requests from the surgeon clear most of the time? Under what circumstances do you need to clarify requests?
   4. When you first started your job, did it take a while to get up to speed with the jargon used in the cardiac surgery ORs here?
   5. Is there a general consensus about when surgeons should arrive in the OR (before incision, after incision, etc)?
      1. And what about when they should leave the OR? Do most stay for a debrief?
   6. Is there a general consensus about how much case-related information team members should review before each case?
   7. Are there any other forms of interaction that you find particularly helpful or unhelpful in the OR?
3. Do you have any final thoughts you would like to share?
4. Who from your team would you recommend we interview?

Thank you for your time today.

***For surgeons only:***

1. *What do they think about these results?*

*[How did they learn to teach?]*

1. To what extent have your thoughts on communication and team dynamics in surgical cases changed over the past year?
2. To what extent do you feel you have been able to (or wanted to!) change any aspects of your communication or leadership style?
   1. What do you hope to continue working on and how?
