# Supplementary material for: Effective Leadership of Surgical Teams: A Mixed Methods Study of Surgeon Behaviors and Functions
Source: Ann Thorac Surg. 2017 Aug;104(2):530–7. doi: 10.1016/j.athoracsur.2017.01.021 (PMC5527126; doi:10.1016/j.athoracsur.2017.01.021)
Supplement: Supplemental Material-E [file mmc6.docx]

Supplemental Material-E: Comparison of leader behavior taxonomy to prior tools

| **Comment Type** | **Description** | **BMRI^1^** | **NOTECHS^2^** | **NOTSS^3^** | **SLI^4^** | **OIORT^5^** | **SGB^6^** |
| --- | --- | --- | --- | --- | --- | --- | --- |
| **Positive Behaviors** | | | | | |  |  |
| ***Elucidator*** |  |  |  |  |  |  |  |
| **Teaching** | Teaching another team member how to do something. Calling attention to something worthy of note to create an opportunity for a team member to learn/ practice. Includes technical teaching, pertaining to science, surgery, medicine, body; and non-technical teaching, pertaining to communication, leadership, teamwork, interactions. | **Not included** | **Leadership and management-leadership** | **Not included** | **Training** | **Instructional behavior-informing; demonstrating technique; directing** | **Teaching-physically show how; Directing-proactively questioning, ask next step** |
| **Constructive criticism** | Providing negative feedback in a constructive manner. | **Not included** | **Teamwork and cooperation-understanding team needs** | **Not included** | **Not included** | **Responding behaviors-give corrective feedback** | **Not included** |
| **Explanation** | Speaking aloud to inform others how he/she perceives what’s going on; thinking or interpreting aloud; providing reasoning for decisions or their outcomes. | **Briefing; information sharing** | **Situation awareness-understand** | **Situation awareness-projecting and anticipating future state; Communication and teamwork-establishing a shared understanding** | **Communi-cating; training; making decisions** | **Instructional behaviors-guiding/advising** | **Teaching-explain thought process and decision** |
| **Relevance giving** | Adding "the because" to requests or interpreting data aloud for others to hear. | **Not included** | **Situation awareness-understand** | **Not included** | **Not included** | **Instructional behaviors-justifying** | **Not included** |
| ***Tone Setter*** |  |  |  |  |  |  |  |
| **Constructive humor** | Making an inclusive joke. Could include self-deprecating humor. | **Information sharing** | **Teamwork and cooperation-team building/maintaining** | **Not included** | **Supporting others** | **Setting tone behaviors-joking positive** | **Not included** |
| **Compliment** | Saying something nice about someone else’s work. | **Not included** | **Not included** | **Not included** | **Not included** | **Responding behaviors-give positive feedback** | **Directing-compliment** |
| **Reassurance** | Saying comforting or forgiving words in response to a mistake made by a team member. | **Not included** | **Teamwork and cooperation-support of others** | **Not included** | **Not included** | **Not included** | **Not included** |
| ***Engagement facilitator*** |  |  |  |  |  |  |  |
| **Collaboration** | Working with another team member to make a decision or interpret data, information, or observations. | **Information sharing** | **Problem solving and decision making-option generation** | **Decision making-considering options; Communication and teamwork-coordinating team activities** | **Making decisions** | **Not included** | **Not included** |
| **Consultation** | Asking another team member if it’s okay to proceed or for a status update from their perspective. | **Inquiry** | **Leadership and management-maintenance of standards; Problem solving and decision making-definition and diagnosis** | **Communication and teamwork-exchanging information** | **Communi-cating** | **Questioning behaviors-open question: action** | **Not included** |
| **Encouragement** | Sharing reassuring words with the room. | **Not included** | **Leadership and management-leadership** | **Leadership-supporting others** | **Supporting others** | **Responding behaviors-encouraging** | **Not included** |
| **Helping/ supporting** | Contributing to the group's work and/or lending assistance to another team member. | **Not included** | **Teamwork and cooperation-support of others** | **Leadership-supporting others** | **Supporting others** | **Not included** | **Not included** |
| **Apology** | Expressing regret for a mistake or a mis-understanding. | **Not included** | **Not included** | **Not included** | **Not included** | **Not included** | **Not included** |
| **Thanks** | Expressing appreciation to another for his/her help or job well done. | **Not included** | **Not included** | **Not included** | **Not included** | **Not included** | **Not included** |
| **Inquiry** | Question to identify potential problem or to indicate concern. | **Inquiry; Vigilance and awareness** | **Problem solving and decision making-definition and diagnosis** | **Situation awareness-gathering information** | **Making decisions** | **Not included** | **Not included** |
| ***Delegator*** |  |  |  |  |  |  |  |
| **Help seeking** | Asking for assistance from others. | **Not included** | **Not included** | **Not included** | **Maintaining standards** | **Not included** | **Not included** |
| ***Safe Space Maker*** |  |  |  |  |  |  |  |
| **Non-surgeon initiated concern** | Team member raises a safety or quality concern. | **Assertion** | **Not included** | **Not included** | **Supporting others** | **Not included** | **Not included** |
| **Non-surgeon initiated questioning** | Team member asks a question without prompting from the surgeon. | **Inquiry** | **Not included** | **Not included** | **Supporting others** | **Responding behaviors-respond to question / comment** | **Directing-respond to resident's request** |
| **Non-surgeon information-sharing** | Team member shares information without prompting from the surgeon. | **Assertion** | **Not included** | **Not included** | **Supporting others** | **Not included** | **Not included** |
| ***Conductor*** |  |  |  |  |  |  |  |
| **Focus returning** | Announcing to the room that it is time to focus on case-related matters. | **Vigilance and awareness** | **Leadership and management-maintenance of standards** | **Leadership-setting and maintaining standards** | **Maintaining standards** | **Not included** | **Not included** |
| **Concern anticipation** | Identifying for team members a likely outcome before it occurs in order to create expectations and alleviate potential worry. | **Contingency management** | **Situation awareness-think ahead** | **Situation awareness-projecting and anticipating future states** | **Coping with pressure** | **Not included** | **Not included** |
| **Step mapping** | Describing in advance for the room to hear the next few steps in the procedure. | **Not included** | **Situation awareness-think ahead** | **Task management-planning and preparation** | **Not included** | **Instructional behavior-warning** | **Not included** |
| **Loop closing for confirmation** | Structured back and forth communication between the surgeon and another team member, in which the surgeon requests a task and the team member repeats to confirm the task has been completed. | **Information sharing** | **Not included** | **Communicating-establishing a shared understanding** | **Communi-cating; maintaining standards** | **Not included** | **Not included** |
| ***Being human*** |  |  |  |  |  |  |  |
| **Self-questioning** | Sharing uncertainty about one's own performance. | **Not included** | **Situation awareness-understand** | **Not included** | **Not included** | **Not included** | **Not included** |
| **Neutral behaviors** | | | | | |  |  |
| ***Tone setter*** |  |  |  |  |  |  |  |
| **Conversation unrelated to the case** | Friendly discussion about personal/non-work related issues, occurring during non-critical periods in the case. | **Information sharing** | **Teamwork and cooperation-team building/maintaining** | **Not included** | **Supporting others** | **Setting tone behaviors-conversing** | **Assisting-general comments not directly related to current case** |
| ***Delegator*** |  |  |  |  |  |  |  |
| **Request** | Giving instruction to a team member to do something. | **Not included** | **Situation awareness-Notice** | **Decision making-selecting and communicating options** | **Directing** | **Not included** | **Directing-verbally direct or redirect** |
| ***Being human*** |  |  |  |  |  |  |  |
| **Showing fatigue** | Showing evidence of stress/strain on the body. | **Not included** | **Not included** | **Not included** | **Not included** | **Not included** | **Not included** |
| **Musing** | Observation of more generalizable nature; may be sarcastic. | **Not included** | **Not included** | **Not included** | **Not included** | **Setting tone behaviors-commenting** | **Not included** |
| **Negative behaviors** | | | | | |  |  |
| ***Elucidator*** |  |  |  |  |  |  |  |
| **Private criticism** | Critiquing or scolding on the side (not for the entire room to hear). | **Not included** | **Not included** | **Not included** | **Not included** | **Not included** | **Not included** |
| **Negative criticism** | Providing negative feedback. | **Not included** | **Teamwork and cooperation-understanding team needs** | **Not included** | **Not included** | **Responding behaviors-give negative feedback** | **Not included** |
| ***Tone setter*** |  |  |  |  |  |  |  |
| **Frustration** | Expressing annoyance or anger. | **Not included** | **Teamwork and cooperation-conflict solving (negative form)** | **Leadership-coping with pressure (negative form)** | **Not included** | **Not included** | **Not included** |
| **Destructive humor** | Sarcasm, demeaning statements, joke at the expense of patient or provider. | **Not included** | **Teamwork and cooperation-team building/maintaining (negative form)** | **Leadership-supportive others (negative form)** | **Supporting others (negative form)** | **Setting tone behaviors-joking negative** | **Not included** |
| ***Conductor*** |  |  |  |  |  |  |  |
| **Need for clarification** | Request by surgeon that prompts a team member to follow up with a repetition of that request, phrased as a question indicating that original request was not understood. | **Not included** | **Leadership and management-planning and preparation (negative form)** | **Communication and teamwork-establishing shared understanding (negative form)** | **Communicating (negative form)** | **Not included** | **Not included** |
| ***Being human*** |  |  |  |  |  |  |  |
| **Jargon** | Speaking in idiosyncratic terms. | **Not included** | **Not included** | **Not included** | **Not included** | **Not included** | **Not included** |
| ***Number of domains not included*** |  | **22** | **12** | **19** | **16** | **19** | **27** |
| ***Number of unobserved domains*** |  | **1 (Briefing)** | **4 (Leadership and management-workload management and authority and assertiveness; Problem solving and decision making-risk assessment and outcome review)** | **3 (Situation awareness-understanding information; Decision making-implementing and reviewing decisions; Task management-flexibility/ responding to change)** | **1 (Managing resources)** | **6 (Instructional behaviors-commanding; Questioning behaviors-closed question, open question, interrogating; Responding behaviors-confirmation; Setting tone behaviors-chastising, insulting; Other)** | **(Teaching-allow resident to learn from "mistake"; Directing-directly point out with instruments or fingers, indirectly point out with camera, direct via teaching medical student; Assisting-direct OR team to support, double check resident's judgment or action, remind, do part of surgeon's job)** |
| ***Explanation for why not observed*** |  | **Taxonomy focuses on phase of the case, not behaviors** | **Taxonomy focuses on phase of the case or functions, not behaviors** | **Taxonomy focuses on functions, not behaviors** | **Taxonomy focuses on functions, not behaviors** | **Taxonomy focuses on teaching, not leadership** | **Taxonomy focuses on guiding residents, not leadership** |
| Notes: [4,27-29] | | | | | | | |
| ^1^ Mazzocco K, Petitti DB, Fong KT, Bonacum D, Brookey J, Graham S, et al. Surgical team behaviors and patient outcomes 2009;197:678–85.  Behavioral Marker Risk Index (BMRI) includes 6 domains: (1) Briefing, i.e., situation/relevant background shared; patient, procedure, site/side identified; plans are stated; questions asked; ongoing monitoring and communication encouraged. (2) Information sharing, i.e., information is shared; intentions are stated; mutual respect is evident; social conversations are appropriate. (3) Inquiry, i.e., asks for input and other relevant information. (4) Vigilance and awareness, i.e., tasks are prioritized; attention is focused; patient/equipment monitoring is maintained; tunnel vision is avoided; red flags are identified. (5) Assertion, i.e., the members of the team are speaking up with their observations and recommendations during critical times. (6) Contingency management, i.e., relevant risks are identified; backup plans are made and executed. | | | | | | | |
| ^2^ Mishra A, Catchpole K, McCulloch P. The Oxford NOTECHS System: reliability and validity of a tool for measuring teamwork behaviour in the operating theatre. Qual Saf Health Care 2009;18:104–8. doi:10.1136/qshc.2007.024760.  Oxford Non-Technical Skills (NOTECHS) system includes 4 domains and a total of 16 subdomains: (1) Leadership and management, (1a) Leadership, i.e., involves/reflects on suggestions/visible/accessible/inspires/motivates/coaches, (1b) Maintenance of standards, i.e., subscribes to standards/monitors compliance to standards/intervenes if deviation/deviates with team approval/ demonstrates desire to achieve high standards, (1c) Planning and preparation, i.e., team participation in planning/plan is shared/understanding confirmed/projects/changes in consultation, (1d) Workload management, i.e., distributes tasks/monitors/reviews/tasks are prioritised/allots adequate time/responds to stress, (1e) Authority and assertiveness, i.e., advocates position/values team input/takes control/persistent/appropriate assertiveness. (2) Teamwork and cooperation, (2a) Team building/maintaining, i.e., relaxed/supportive/open/inclusive/polite/friendly/use of humour/does not compete, (2b) Support of others, i.e., helps others/offers assistance/gives feedback, (2c) Understanding team needs, i.e., listens to others/recognises ability of team/condition of others considered/gives personal feedback, (2d) Conflict solving, i.e., keeps calm in conflicts/suggests conflict solutions/concentrates on what is right. (3) Problem-solving and decision-making, (3a) Definition and diagnosis, i.e., uses all resources/analytical decision-making/reviews factors with team, (3b) Option generation, i.e., suggests alternative options/asks for options/reviews outcomes/confirms options, (3c) Risk assessment, i.e., estimates risks/considers risk in terms of team capabilities/estimates patient outcome, (3d) Outcome review, i.e., reviews outcomes/reviews new options/objective, constructive and timely reviews/makes time for review/seeks feedback from others/conducts post-treatment review. (4) Situation awareness, (4a) Notice, i.e., considers all team elements/asks for or shares information/aware of available of resources/encourages vigilance/checks and reports changes in team/requests reports/updates, (4b) Understand, i.e., knows capabilities/cross-checks above/shares mental models/speaks up when unsure/updates other team members/discusses team constraints, (4c) Think ahead, i.e., identifies future problems/discusses contingencies/anticipates requirements. | | | | | | | |
| ^3^ Yule S, Flin R, Paterson-Brown S, Maran N, Rowley D. Development of a rating system for surgeons' non-technical skills. Med Educ 2006;40:1098–104. doi:10.1111/j.1365-2929.2006.02610.x.  Non-Technical Skills for Surgeons (NOTSS) rating system includes 5 domains: (1) Situation awareness, i.e., gathering information, understanding information, projecting and anticipating future state. (2) Decision making, i.e., considering options, selecting and communicating option, implementing and reviewing decisions. (3) Task management, i.e., planning and preparation, flexibility ⁄ responding to change. (4) Leadership, i.e., setting and maintaining standards, supporting others, coping with pressure. (5) Communication and teamwork, i.e., exchanging information, establishing a shared understanding, co-ordinating team activities. | | | | | | | |
| ^4^ Parker SH, Flin R, McKinley A, Yule S. The Surgeons’ Leadership Inventory (SLI): a taxonomy and rating system for surgeons” intraoperative leadership skills. The American Journal of Surgery 2013;205:745–51. doi:10.1016/j.amjsurg.2012.02.020.  Surgeons' Leadership Inventory (SLI) system includes 8 domains: (1) Maintaining standards, i.e., supporting safety and quality by adhering to acceptable principles of surgery, following codes of good clinical practice, and enforcing theater procedures and protocols by consistently demonstrating appropriate behaviors (i.e., asking for help when required). (2) Making decisions, i.e., seeking out appropriate information and generating alternative possibilities or courses of action, synthesizing the information, choosing a solution to a problem, and letting all relevant personnel know the chosen option; making an informed prompt judgment on the basis of information, clinical situation, and risk and continually reviewing its suitability in light of changes in the patient's condition. (3) Managing resources, i.e., assigning resources (people and equipment) depending on the situation or context; delegating tasks appropriately to team members, and ensuring the team has what it needs to accomplish the task. (4) Directing, i.e., clearly stating expectations regarding accomplishment of task goals; giving clear instructions; using authority where required; demonstrating confidence in both leadership and technical ability. (5) Training, i.e., instructing and coaching team members according to goals of the task; modifying own behavior according to team's educational needs; identifying and maximizing educational opportunities. (6) Supporting others, i.e., judging the capabilities of team members; offering assistance where appropriate; establishing a rapport with team members and actively encouraging them to speak up. (7) Communicating, i.e., giving and receiving information in a timely manner to aid establishment of a shared understanding among team members; speaking appropriately for the situation; asking for input from team members. (8) Coping with pressure, i.e., showing flexibility and changing plans if required to cope with changing circumstances to ensure that goals are met; anticipating possible complications and communicating them to staff; adopting a forceful manner if appropriate without undermining the role of other team members. | | | | | | | |
| ^5^ Hauge LS, Wanzek JA, Godellas C. The reliability of an instrument for identifying and quantifying surgeons' teaching in the operating room. The American Journal of Surgery 2001;181:333–7.  Observation Instrument for Operating Room Teaching face validity identifies teaching behaviors in four categories: informing*, questioning*, responding*, and setting tone*, and 26 subcategories of specific teaching behaviors: (1) Instructional behaviors include Informing: basic science* (i.e., providing basic science information or background), Informing: surgical technique* (i.e., providing information about specific surgical techniques), Informing: patient history (i.e., providing information about patient history, background), Informing: other (i.e., providing information about history, trivia, etc.), Demonstrating technique (i.e., demonstrating surgical technique or procedure), Guiding/advising* (i.e., verbal guidance of a surgical technique, giving choice), Justifying* (i.e., providing justification for surgical decisions), Warning* (i.e., providing warning about decision/move before it takes place), Directing* (i.e., verbal direction of a surgical technique, giving no choice), Commanding (i.e., verbal direction with fierce tone); (2) Questioning behaviors include Closed question*(i.e., asking a yes/no question), Open question: action (i.e., asking open-ended question about upcoming action/task), Open question: information (i.e., asking open-ended question about what person knows), Interrogating (i.e., asking series of questions with a tone of intimidation); (3) Responding behaviors include Encouraging (i.e., cheering on a resident’s action or response), Confirming* (i.e., confirming a resident’s choice of action or surgical move), Give specific positive feedback (i.e., providing specific positive feedback about decision/move), Give specific negative feedback* (i.e., providing specific negative feedback about decision/move), Give corrective feedback (i.e., providing specific corrective feedback about decision/move), Respond to question/comment* (i.e., responding to resident/student question or comment); and (4) Setting tone behaviors include Commenting* (i.e., making statement about procedure or environment), Joking positive (i.e., jokes that add levity to environment w/out personal insult), Joking negative (i.e., jokes that are discouraging, disparaging, insulting), Conversing* (i.e., eliciting conversation about topics other than operation), Chastising (i.e., severe criticism of others’ action or decision), Insulting (i.e., treating an individual with contempt); and Other (i.e., behavior/action that does not fall in any of the above). Categories marked with * showed reliability. | | | | | | | |
| ^6^ Chen XP, Williams RG, Sanfey HA, Smink DS. A taxonomy of surgeons’ guiding behaviors in the operating room. The American Journal of Surgery 2015;209:15–20. doi:10.1016/j.amjsurg.2014.07.018.  Surgeon Guiding Behaviors identified 3 guiding behaviors (16 subcategories): (1) Teaching (i.e., intending to teach the resident something new by showing how to perform the current operation, by explaining the surgeon’s thought process, and/or by adding new knowledge about instruments for future operation); (2) Directing (i.e., intending to navigate the resident based on his/her level of knowledge and skills through the operation directly or indirectly toward the successful completion of the operation); and (3) Assisting: the intention of these guiding behaviors is to support the resident to complete the task by facilitating the OR team to support, by reassuring the resident’s intraoperative judgments, or through performing in accordance with the resident’s decisions). | | | | | | | |
