# Supplementary figures and images for: Effective Leadership of Surgical Teams: A Mixed Methods Study of Surgeon Behaviors and Functions
Source: Ann Thorac Surg. 2017 Aug;104(2):530–7. doi: 10.1016/j.athoracsur.2017.01.021 (PMC5527126; doi:10.1016/j.athoracsur.2017.01.021)

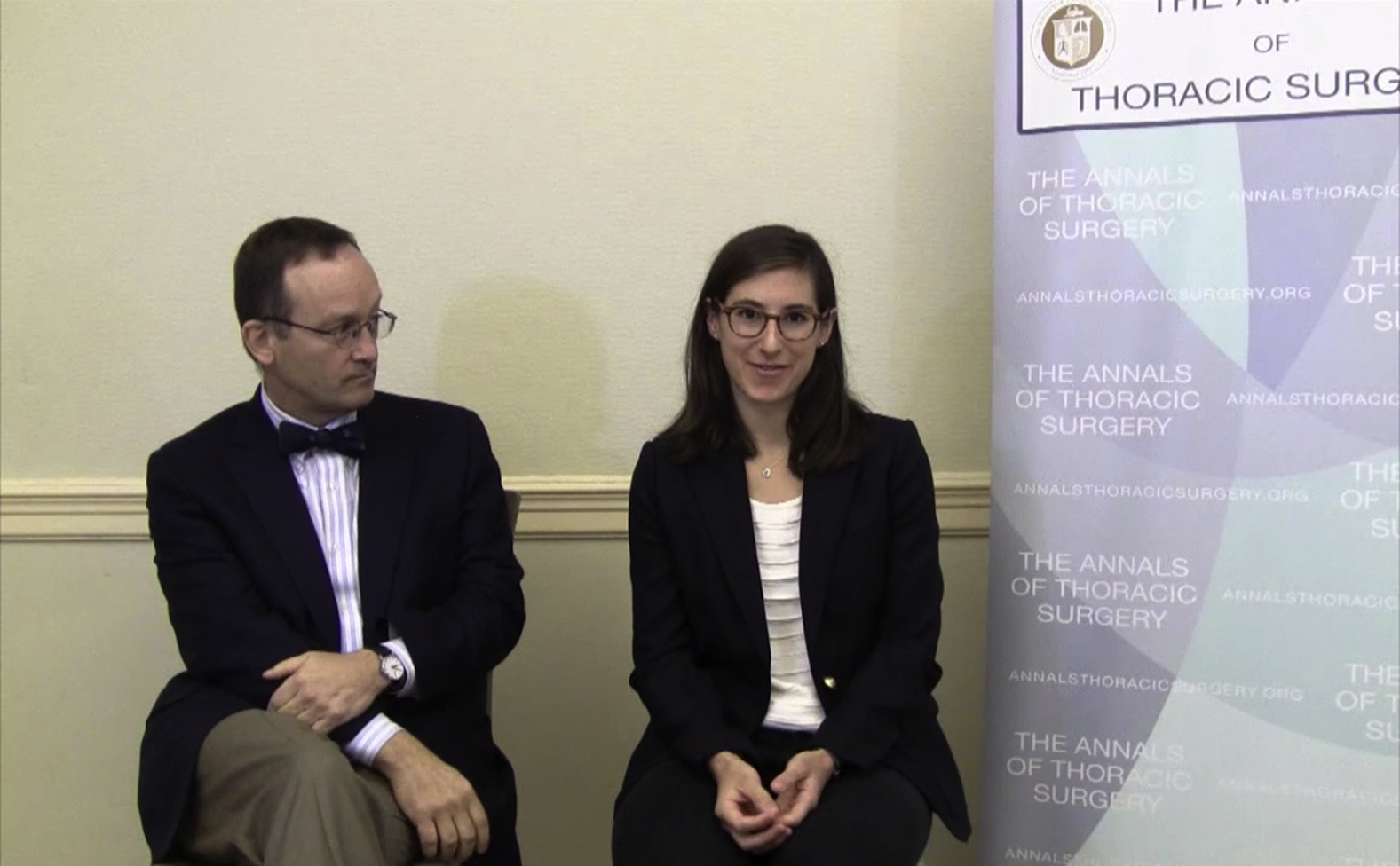

Supplement: Author Interview [file mmc7.jpg]
